# A high efficiency active X^2^G boost converter with hybrid optimized proportional integral controller for PV powered EV charging applications

**DOI:** 10.1038/s41598-025-20320-2

**Published:** 2025-10-16

**Authors:** G. Chandrasekar, S. A. Lakshmanan

**Affiliations:** Department of Electronics and Communication Engineering, Amrita School of Engineering, Amrita Vishwa Vidyapeetham, Chennai, India

**Keywords:** Photovoltaic system, Electric vehicles, Meerkat optimization algorithm, Siberian tiger optimization, DC–DC converter, Engineering, Electrical and electronic engineering

## Abstract

Photovoltaic (PV) integration with Electric Vehicles (EV) plays a pivotal role in promoting sustainable transportation and fostering clean energy ecosystems. However, the development of high-efficiency, high-gain power converters and robust control strategies remains a critical challenge. This paper presents an innovative approach to enhancing PV based EV charging systems through the development of a novel Active X^2^G Boost Converter, which uniquely integrates a quasi Z-source network (qZSN) with a voltage multiplier stage in a single-switch configuration. This design significantly improves voltage gain, energy conversion efficiency and reduces component stress, addressing key limitations in existing converter topologies. A standout feature of the proposed system is the implementation of a Hybrid Siberian Tiger–Meerkat Optimized Proportional-Integral (HSTM-PI) controller, a newly introduced metaheuristic control strategy that blends the global search capability of Meerkat Optimization Algorithm (MOA) with local refinement strength of the Siberian Tiger Optimization (STO). This hybridization ensures faster convergence, enhanced dynamic voltage regulation and greater control robustness under fluctuating solar and load circumstances. The system also incorporates a bidirectional converter, battery storage, and grid synchronization through a three-phase inverter. Comprehensive mathematical modelling, MATLAB simulation and experimental validation confirm the system’s effectiveness, achieving a notable 96% efficiency, outperforming conventional designs in both regulation precision and operational reliability.

## Introduction

**Fig. 1:**
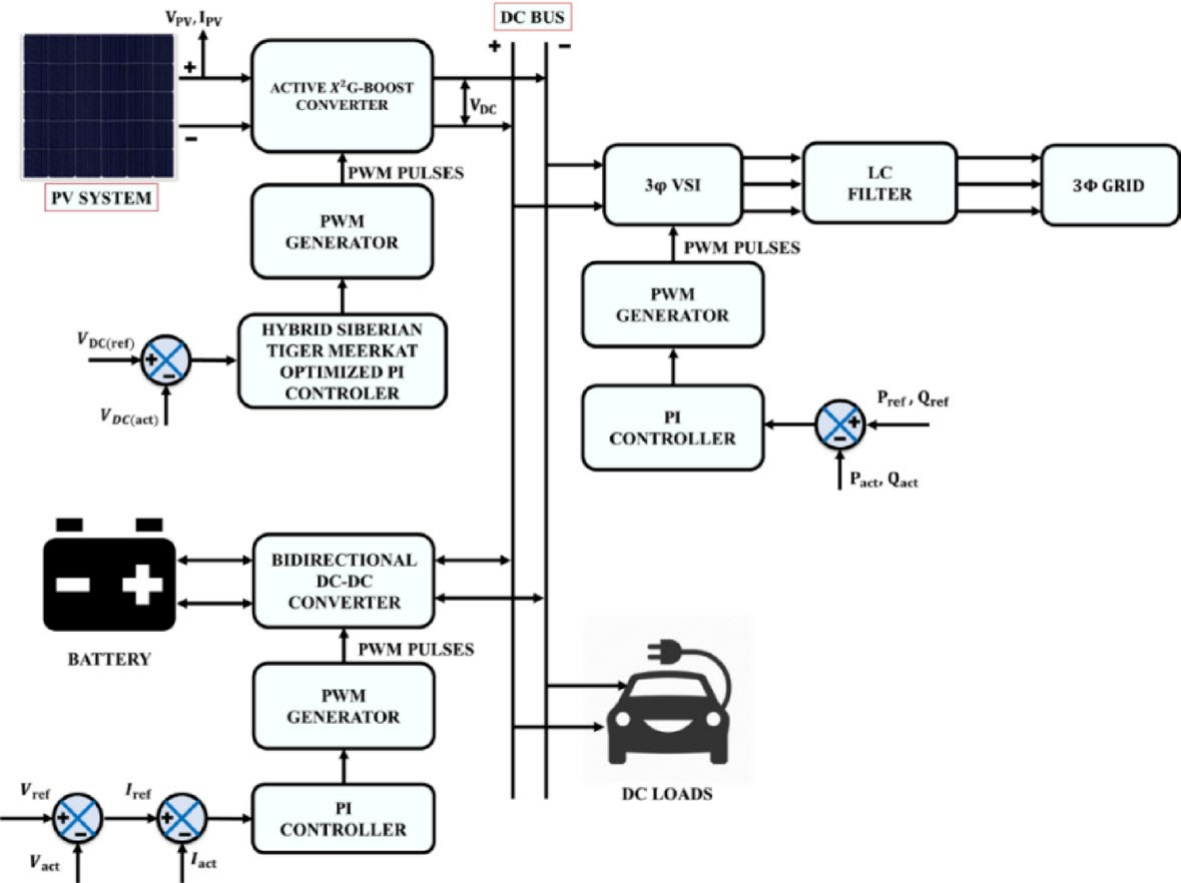
Generalized block diagram PV powered EV system with three-phase electric grid and bi-directional converter.

Energy generation and consumption of any country typically increases with the growth of its population and economy. Depletion of fossil fuels such as coal, natural gas, petroleum, and their harmful effects on the environment have made it important for nations to shift their attention towards utilizing the energy from Renewable Energy Sources (RES)^1^. Among various RES, solar PV system stands out as a primary source^2^. Integration of PV with EV plays a crucial part in enhancing energy ecosystems and sustainable transportation^3^. This combined approach helps to exploit the PV energy for powering EVs, providing a comprehensive solution to address the environmental, economic, and societal challenges globally. Moreover, the flexibility and advancement in PV technology facilitate the establishment of PV fed EV charging stations in various locations, in particular homes, businesses, and public areas to access by the users conveniently. Therefore, solar PV integrated EV system promotes the availability of eco-friendly transportation solutions while supporting the security, offers an effective pathway to decrease grid dependency and viability of energy networks^4^. However, adoption of more EV places additional strain on the power grid, especially as daytime charging intensifies peak demand. A generalized block diagram of the PV powered EV system with three-phase electric grid and bi-directional converter is shown in Fig. 1.

In PV-based EV charging stations, achieving high voltage gain is critical to efficiently step up the low and variable DC output of PV panels to levels appropriate for charging batteries or interfacing with the DC bus^5^. Conventional converters such as the Boost, Buck-Boost, SEPIC, Cuk, Zeta and Luo topologies are widely adopted, while they inherently suffer from limitations in voltage gain and efficiency at high duty cycles^6^. Specifically, Boost converter experiences severe losses and reduced efficiency when operating with duty ratios approaching unity^7^. Buck-Boost, Cuk and Zeta converters exhibit inverting characteristics or complex control requirements and the SEPIC converter suffers from high voltage stress and limited gain^8,9^. Similarly, Luo convertersthough providing improved ripple performance, shows practical limitations due to reduced power density^10^. To address these challenges, several topologies have been developed. Cascaded Boost Converter (CBC)^11^ connects multiple boost stages in series to achieve higher voltage gain. Nevertheless, this advantage is offset by increased switching losses, control complexity and reduced overall efficiency. Quadratic Boost Converter (QBC)^12^ offers enhanced voltage gain using a dual inductor-capacitor structure, but it introduces additional passive components,increases the system size and cost.

Interleaved converters with parallel multiple switching phasesimprove the input current ripple and thermal distribution. However, this converters suffer from control complexity and component mismatch issues^13^. Meanwhile, coupled inductorbased topologies provide significant gain with reduced switch stress, but introduce leakage inductance and insulation complexities that degrade efficiency and reliability^14^. A feasible replacement lies in impedance network integration within power converter architecture^15,16^. Impedance networks, particularly those utilizing inductors and capacitors in specific configurations, serve to boost the voltage, provide shoot-through protection, and ensure continuous current flow. These networks play a significant role in achieving voltage gain without relying on extreme duty cycles, thereby enhancing efficiency and reducing Electro Magnetic Interference (EMI)^17–19^.

**Fig. 2:**
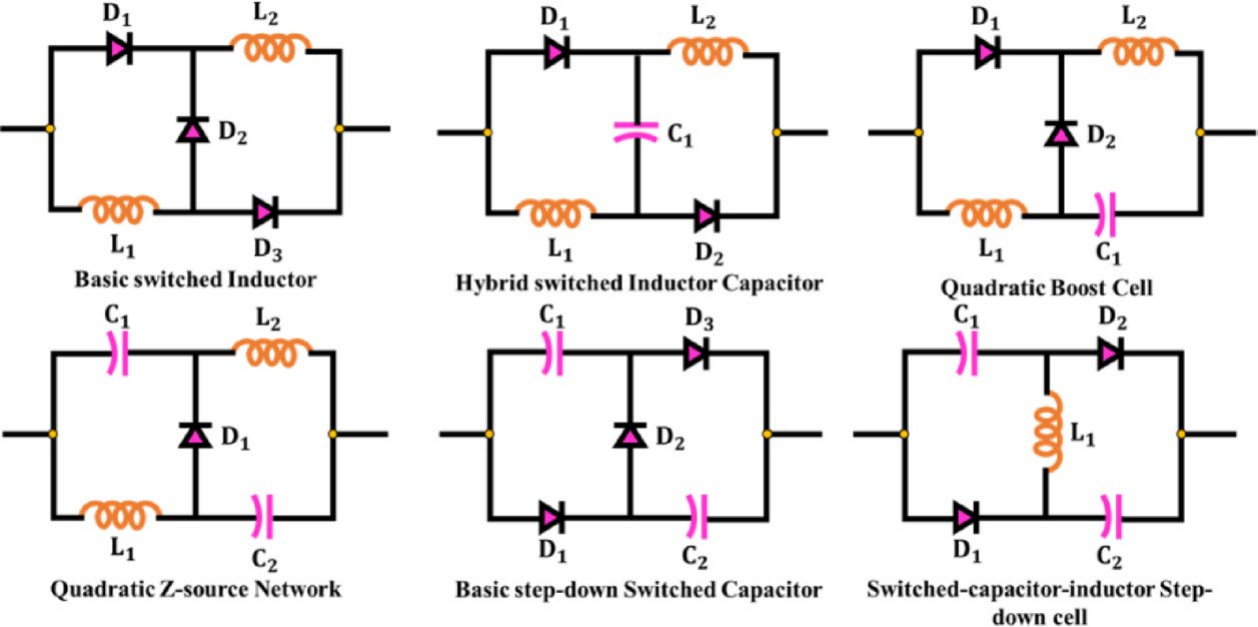
Different impedance network topologies.

Figure 2 illustrates a range of impedance network topologies that have been explored to improve the DC–DC conversion performance. While each offers specific advantages in voltage lift or ripple control, they often suffer from either increased component count, high switch stress or non-ideal shoot-through handling. Among these, qZSN^20^ emerges as the most suitable choice for PV powered EV applications. Unlike traditional Z-source networks, the qZSN maintains continuous input current, significantly reduces inrush current during startup, and enables Boost and Buck operation within the same topology^21^. Furthermore, it facilitates shoot-through state utilization, which enhances reliability and protects against partial shading conditions in PV arrays^22^.

The step-up boost converter with voltage multiplier cell based on a three-winding coupled inductor offers several advantages, making it highly suitable for applications requiring elevated DC output from low-voltage sources. The voltage multiplier cell further increases gain while maintaining continuous input current and improving power transfer capability. Moreover, the use of a three-winding coupled inductor provides magnetic integration, which helps reduce the overall converter size and component count. However, these benefits come with certain drawbacks. The design involves complex magnetic coupling, which may lead to leakage inductance, voltage spikes, and EMI issues if not carefully managed. Also, component design and control are more challenging, requiring precise winding turns ratio and careful layout to avoid losses or instability ^23^. Implementing high-gain interleaved converter of non-isolated type offers a combination of high performance, adaptability, and efficiency.

The interleaved topology reduces input current ripple, improves thermal distribution across switches, and enhances dynamic response making it ideal for fuel cells, which are sensitive to ripple and require smooth power delivery. The high-gain capability allows the converter to effectively boost the low output to the level required by the electric drive train without resorting to extreme duty cycles. However, increased complexity of control and gate driving circuits is noticed ^24^. The ultrahigh step-up converter offers significant advantages that require high voltage conversion from low-voltage sources. The use of a three-winding coupled inductor enables extremely high voltage gain without demanding high duty cycles, while the switched capacitor network further boosts the output voltage efficiently. However, the complexity of the magnetic design can introduce issues such as leakage inductance and magnetic interference, which may affect performance and require snubber circuits or clamping techniques ^25^.

While advanced converters enhance the electrical functioning of PV-based EV charging stations. Overall system stability and voltage regulation critically depend on the design of an effective control mechanism. A key requirement in such systems is a robust voltage controller that regulates the output voltage under dynamic operating conditions^26^. The Proportional-Integeral (PI) controller remains one of the most widely used control strategies in Power Electronics (PE) due to its simplicity, ease of implementation and adequate performance in linear and nearsteady-state conditions^27^. It operates by minimizing the error between the reference and actual voltage, thus ensuring regulated output. However, the effectiveness of a conventional PI controller is largely constrained by its fixed gain parameters, which are often manually tuned. Static tuning leads to sub-optimal performance when the system operates in nonlinear or time-varying environments, which is a common scenarios in renewable energy applications. Poorly tuned gains results in overshoot, increased settling time or even instability under fast dynamic conditions^28^. To overcome these limitations, researchers have increasingly adopted metaheuristic optimization algorithms to automatically tune the PI controller gains.

Conventional techniques such as Genetic Algorithm (GA), Particle Swarm Optimization (PSO), Cuckoo Search Algorithm (CSA), Whale Optimization Algorithm (WOA) and Artificial Bee Colony (ABC) algorithm have been employed to optimize the performance of PI controllers by minimizing errorbased objective functions^29,30^. While these algorithms have shown improved tuning over manual approaches, each has inherent limitations. GA and PSO suffer from premature convergence and slow exploration in high-dimensional search spaces. CSA and WOA stagnate in local minima when diversity among the population diminishes and ABC, while good at exploration, lacks sufficient exploitation ability to fine-tune solutions effectively. In recent years, Siberian Tiger Optimization (STO) and Metaheuristic Optimization Algorithms (MOA) have recently gained attention due to their superior convergence characteristics. The STO^31^ exhibits strong local exploitation abilities through its prey-hunting and bear-fighting strategies, allowing it to refine candidate solutions effectively. On the other hand, MOA^32^ introduces diverse exploratory behaviors such as outward and cooperative search modes, as well as emergency escape mechanisms like fleeing and fighting when facing local stagnation. While each algorithm demonstrates specific strengths, their individual limitations still pose challenges in balancing exploration and exploitation.

To address this trade-off, research work carried out introduces a novel converter topology, termed Active X^2^G-Boost converterwhich integrates a qZSN with a voltage multiplier stage and operates using a single-switch configuration to enhance voltage amplification and efficiency. Moreover, this research paper proposes the hybridization of STO and MOA techniques i. e Hybrid Siberian Tiger-Meerkat (HSTM) optimized PI controller to ensure the voltage stability of the proposed Active X^2^G-Boost Converter. By MOA during the early phase and integrating the intensification capability of STO in the later stages, the HSTM algorithm achieves a more balanced and dynamic tuning approach. This hybrid algorithm not only avoids premature convergence but also ensures faster and more accurate identification of optimal PI gain values, leading to enhanced dynamic response, reduced steady-state error, and improved robustness of the propsed Active X^2^ G-Boost converter control system. The propsoed HSTM optimized PI controller is employed for precise voltage regulation under varying PV and load conditions. The system also incorporates a bidirectional converter interfaced with a battery storage unit to manage energy flow and improve system reliability. Furthermore, grid synchronization is achieved via a three-phase Voltage Sourcce Inverter (VSI) and filter. The proposed Active X^2^G Boost Converter along with the HSTM-PI controller is mathmatically modelled and performance is evaluated under steady state and dynamic conditions. The propsoed architecture is validated through extensive MATLAB simulations and experimental laboratory implementation. Major contributions of the proposed research work carriedout in this paper are summerized as follows.

- Active X^2^G Boost Converter is proposed that combines a qZSN and a voltage multiplier using a single switch, offering high voltage gain, low losses, and improved performance for solar-powered EV charging systems.
- Based on hybrid optimization, HSTM-PI controller is implemented that improves voltage regulation, reduces overshoot, and enhances stability of the converter under PV source interuption and dynamic evnironmental conditions.
- Built and validated a Active X^2^G Boost Converter with HSTM-PI controller for PV based EV charging system that integrates solar panels, battery storage, and grid connectionachieving 96% efficiency in both simulation and experimental analysis.

This paper is organized as follows. Mathematical modelling of the proposed Active X^2^G Boost Converter is discussed in Section "Mathematical modelling of proposed active X2G boost converter". In section "Mathematical modelling of proposed hybrid Siberian Tiger-Meerkat optimized PI controller", Modelling of HSTM-PI controller implemented for this proposed study is analyzed. Bi-directional DC–DC converter with battery and grid voltage synchronization are explored in section "Modelling of bidirectional converter with battery". Section "Results and discussion" presents the simulation results and experimental laboratory prototype implementation to validate the performance of the proposed research work, along with a comparative analysis against existing methods. Finally, this research paper is concluded in section "Conclusions".

## Mathematical modelling of proposed active X^2^G boost converter

**Fig. 3:**
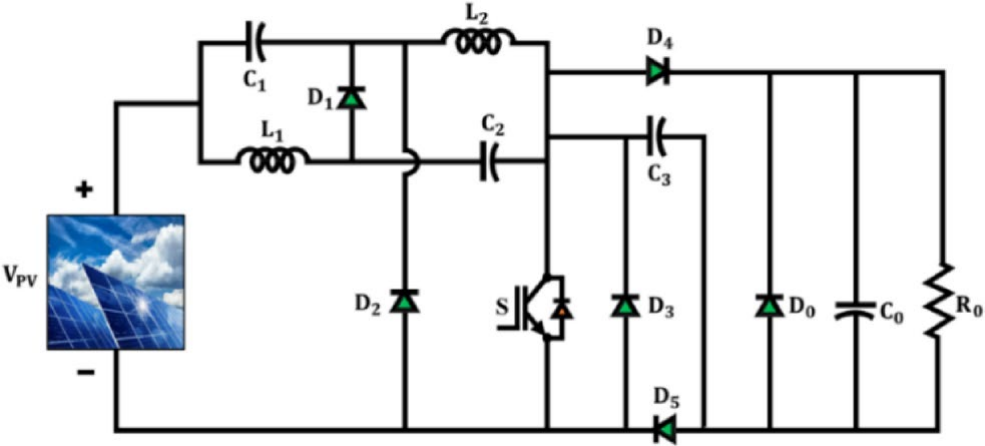
Architecture of active proposed Active X^2^G Boost converter.

The proposed Active ${X}^{2}$G Boost converter is modelled to provide high voltage amplification and augmented energy conversion efficiency. Its core design integrates qZSN with Voltage multipier stage, which is controlled using a single-switch topology as illustrated in Fig. 3. The components ${C}_{1},{C}_{2},{L}_{1},{L}_{2}$ and ${D}_{1}$ form the qZSN, which provides continuous input current and inherent shoot-through immunity. The cascaded configuration of capacitor ${C}_{3}$ and diodes ${D}_{3}$ and ${D}_{5}$ form the voltage multiplier stag and which enables the converter to step up low PV voltages to higher levels by reducing voltage stress on components and enhancing the voltage gain. Unlike prior designs that often employ multiple switches or complex control schemes, this architecture achieves high voltage gain using a single-switch topology, significantly reducing control complexity, switching losses and cost. The qZSN ensures continuous input current and shoot-through immunity, while the voltage multiplier stage uniquely integrated with capacitors and diodes in a cascaded form amplifies the output voltage without increasing component stress. A key technical contribution is the optimized arrangement of the qZSN and the voltage multiplier that enables reduced inrush current, improved electromagnetic compatibility and lower voltage stress across the switch and diodes. Moreover, the converter supports stable operation under a wide duty cycle range without entering saturation or exhibiting control instability.

By incorporating a single-switch configuration, the converter substantially reduces switching frequency and current path resistance, thereby minimizing switching and conduction losses. The converter functions with two distinct operational modes per switching cycle under CCM.

### Mode 1 operation

**Fig. 4:**
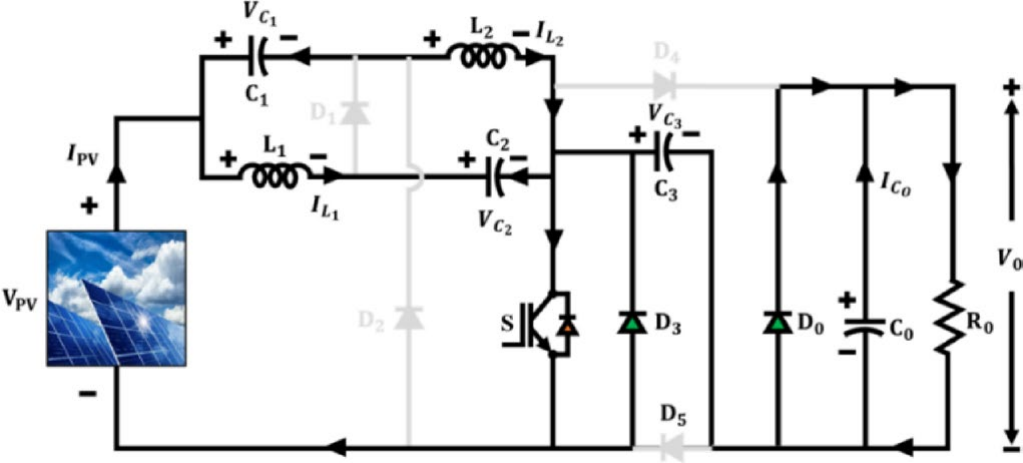
Mode 1 operation of proposed Active X^2^G Boost converter.

In mode 1 operation of proposed Active X^2^G Boost converter in Fig. 4, the switch is ON and inductors ${L}_{1}$, ${L}_{2}$ are in the state of charging, while the capacitors ${C}_{1}$ and ${C}_{2}$ are in the state of discharging. Moreover, in this switching state, diode ${D}_{4}$ is reverse biased, which in turn allows the isolation of output from the input. The diodes ${D}_{1},{D}_{2}$ and ${D}_{5}$ are also reverse biased during this operation. On applying KVL,

1 $$V_{PV} - V_{{L_{1} }} + V_{{C_{2} }} = 0 \Rightarrow V_{{L_{1} }} = V_{PV} + V_{{C_{2} }}$$

2 $$V_{PV} + V_{{C_{1} }} - V_{{L_{2} }} = 0 \Rightarrow V_{{L_{2} }} = V_{PV} + V_{{C_{1} }}$$

3 $${V}_{{C}_{o}}={V}_{{R}_{o}}={V}_{o}$$

### Mode 2 operation

**Fig. 5:**
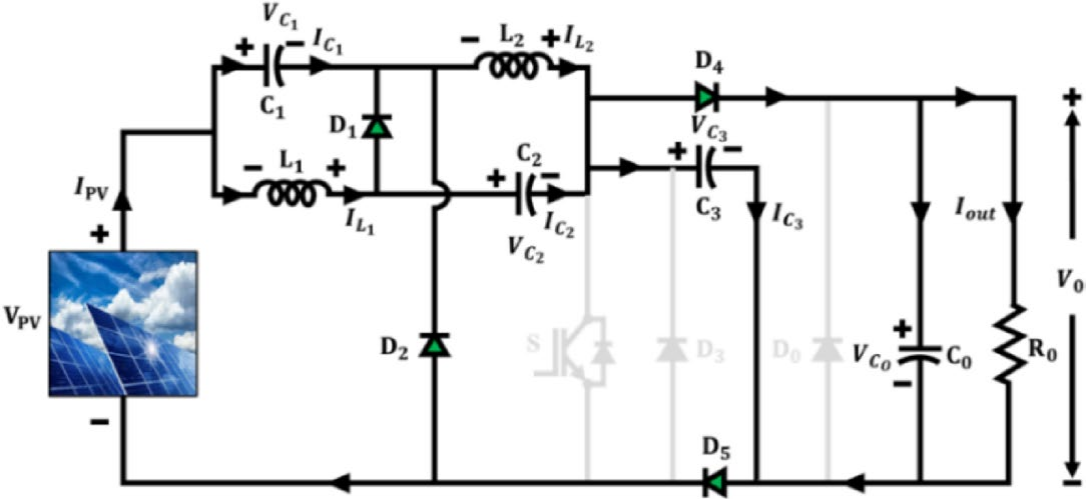
Mode 2 operation of proposed Active X^2^G Boost converter.

Mode 2 operation of proposed Active X^2^G Boost converter is illustrated in Fig. 5. The switch is turned OFF and inductors ${L}_{1}, {L}_{2}$ discharge their stored energy, while the capacitors ${C}_{1}$ and ${C}_{2}$ are in the state of charging. As opposed to mode 1, the conduction of diode ${D}_{4}$ prevents the input side from being isolated from the external converter circuit.

**Fig. 6:**
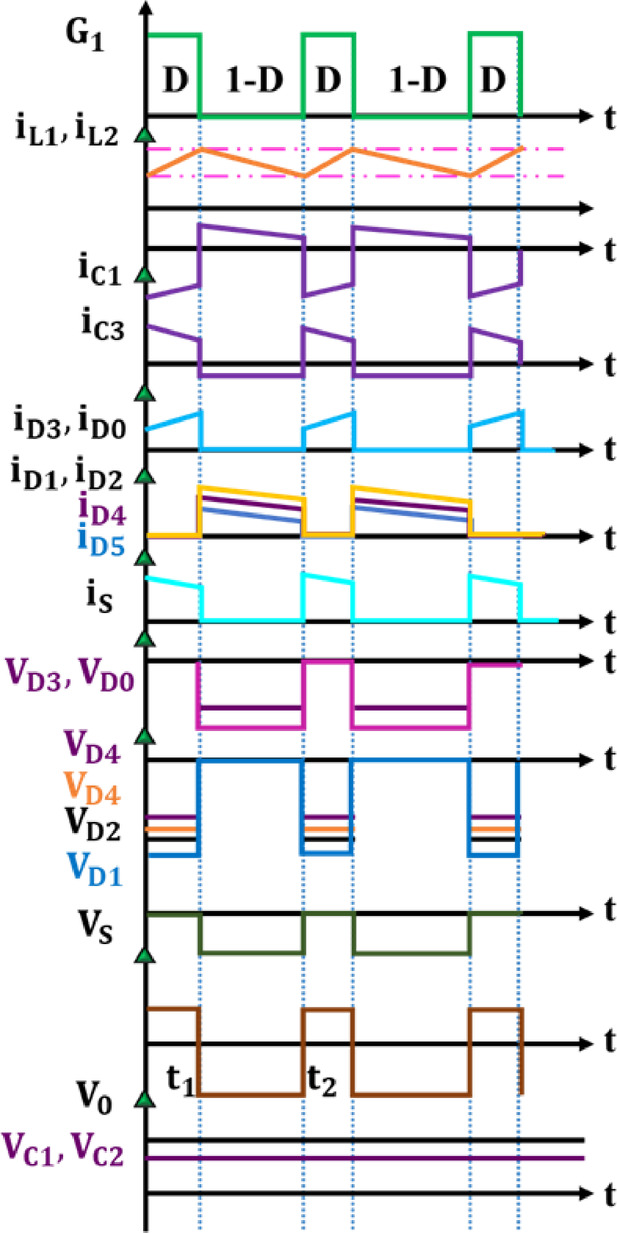
Switching pulse and operational waveform of Active X^2^G Boost converter.

The diodes ${D}_{1},{D}_{2}$ and ${D}_{5}$ becomes forward biased and are in the conducting state during this phase. Switching pulse and operational waveform of the proposed Active X^2^G Boost converter is shown in Fig. 6. Applying KVL,

4 $${V}_{PV}={V}_{{C}_{1}}$$

5 $${V}_{PV}-{V}_{{L}_{1}}-{V}_{{C}_{2}}-{V}_{{C}_{3}}=0$$

6 $$V_{{L_{2} }} - V_{{C_{3} }} = 0 \Rightarrow V_{{L_{2} }} = V_{{C_{3} }}$$

On substituting Eq. (6) in (5),

7 $${V}_{PV}-{V}_{{L}_{1}}-{V}_{{L}_{2}}-{V}_{{C}_{2}}=0$$

8 $${V}_{{C}_{o}}+{V}_{{C}_{3}}=0$$

9 $${V}_{{C}_{3}}=-{V}_{{C}_{o}}=-{V}_{o}$$

### Voltage gain evaluation

To ensure steady-state current in inductors, volt-second balance condition is applied and the resultant equation is given as

10 $${V}_{L(ON)}\cdot D+{V}_{L\left(OFF\right)}\cdot \left(1-D\right)=0$$

For inductor ${L}_{1}$, ${V}_{{L}_{1}(ON)}={V}_{PV}+{V}_{{C}_{2}}$ and ${V}_{{L}_{1}(OFF)}={V}_{PV}-{V}_{{C}_{2}}-{V}_{{C}_{3}}$. On applying volt-second balance law to ${L}_{1}$, with ON and OFF state voltages substituted is given as,

11 $$D\left({V}_{PV}+{V}_{{C}_{2}}\right)+\left(1-D\right)\left({V}_{PV}-{V}_{{C}_{2}}-{V}_{{C}_{3}}\right)=0$$

On substituting $${V}_{{C}_{3}}=-{V}_{o}$$,

12 $$D\left({V}_{PV}+{V}_{{C}_{2}}\right)+\left(1-D\right)\left({V}_{PV}-{V}_{{C}_{2}}+{V}_{o}\right)=0$$

For inductor ${L}_{2}$, ${V}_{{L}_{2}(ON)}={V}_{PV}+{V}_{{C}_{1}}$ and ${V}_{{L}_{2}(OFF)}={V}_{{C}_{3}}$. On applying volt-second balance law on ${L}_{2}$,

13 $$D\left({V}_{PV}+{V}_{{C}_{1}}\right)+\left(1-D\right)\left(-{V}_{o}\right)=0$$

On solving the above equation,

14 $${V}_{o}=\frac{D}{1-D}\left({V}_{PV}+{V}_{{C}_{1}}\right)$$

On assuming ${V}_{{C}_{1}}\approx {V}_{{C}_{2}}={V}_{C}$, Eq. (12) becomes,

15 $$D\left({V}_{PV}+{V}_{C}\right)+\left(1-D\right)\left({V}_{PV}-{V}_{C}+{V}_{o}\right)=0$$

On substituting Eq. (14) in (15),

16 $$D\left({V}_{PV}+{V}_{C}\right)+\left(1-D\right)\left({V}_{PV}-{V}_{C}+\frac{D}{1-D}\left({V}_{PV}+{V}_{C}\right)\right)=0$$

On solving the above equation,

17 $$\frac{{V}_{o}}{{V}_{PV}}=\frac{2D}{1-D}$$

### Voltage and current stress derivations

Accurate estimation of voltage and current stresses on power components is critical for optimizing converter performance, efficiency, and thermal reliability. When $S$ is OFF, maximum voltage appears across the switch is expressed as

18 $${V}_{S}={V}_{{C}_{2}}+{V}_{{C}_{3}}+{V}_{{C}_{0}}\approx {V}_{o}={V}_{PV}\cdot \frac{2D}{1-D}$$

Switch current stress during ON is the sum of inductor currents with ripple,

19 $${I}_{S}={I}_{{L}_{1}}+{I}_{{L}_{2}}={I}_{out}\cdot \left(\frac{1-D}{2D}\right)+\frac{\Delta {I}_{{L}_{1}}}{2}+\frac{\Delta {I}_{{L}_{2}}}{2}$$

The average output current is calculated using Ohm’s law and gain,

20 $${I}_{out}=\frac{{V}_{o}}{{R}_{load}}=\frac{2D}{1-D}\cdot \frac{{V}_{PV}}{{R}_{Load}}$$

Voltage stress of diodes ${D}_{1},{D}_{2},{D}_{5}$,

21 $${V}_{{D}_{1}}={V}_{{D}_{2}}={V}_{{D}_{5}}={V}_{PV}$$

Voltage stress of diode ${D}_{4}$ is,

22 $${V}_{{D}_{4}}={V}_{PV}\cdot \frac{2D}{1-D}$$

Current stress of diodes ${D}_{4},{D}_{0}$ is,

23 $${I}_{{D}_{4}}={I}_{{D}_{0}}={I}_{out}=\frac{2D}{1-D}\cdot \frac{{V}_{PV}}{{R}_{load}}$$

Ripple current through each inductor is expressed as,

24 $$\Delta {i}_{L}=\frac{\left({V}_{PV}+{V}_{C}\right)\cdot D\cdot {T}_{S}}{L}$$

On assuming ${V}_{C}\approx {V}_{PV}$, the ripple simplifies to,

25 $$\Delta {i}_{L}=\frac{2{V}_{PV}\cdot D\cdot {T}_{S}}{L}$$

Peak inductor current includes ripple added to average,

26 $${I}_{Lpeak}={I}_{Lavg}+\frac{\Delta {I}_{L}}{2}$$

Average inductor current is approximately half the output current,

27 $${I}_{Lavg}\approx \frac{{I}_{out}}{2}=\frac{D}{1-D}\cdot \frac{{V}_{PV}}{{R}_{load}}$$

Ripple voltage across each capacitor is defined as,

28 $$\Delta {V}_{C}=\frac{{I}_{C}\cdot D\cdot {T}_{s}}{C}$$

The required inductance value is calculated to limit the inductor current ripple within a desired threshold during the switching period,

29 $$L=\frac{\left({V}_{PV}+{V}_{{C}_{1}}\right)\cdot D}{\Delta {i}_{L}\cdot {f}_{s}}$$

To maintain output voltage stability and suppress ripple, the minimum capacitance is determined based on allowable voltage ripple specifications,

30 $$C=\frac{{I}_{C}\cdot D}{\Delta {v}_{C}\cdot {f}_{s}}$$

The proposed converter employs an HSTM-PI controller to maintain voltage regulation and mitigate dynamic disturbances, thereby ensuring reliable steady-state and transient performance. Modelling of HSTM-PI controller is discussed in the following section.

### State Space averaging model of the proposed converter

Assume that the converter operates in steady-state under CCM. The inductor currents ${i}_{L1}, {i}_{L2}$ and capacitor voltages ${v}_{C1}, {v}_{C2}, {v}_{C3}, {v}_{C0}$ are considered state variables and the control input is the duty cycle D. During mode 1,

31 $$\frac{d{i}_{L1}}{dt}=\frac{{V}_{PV}+{v}_{C2}}{{L}_{1}}$$

32 $$\frac{d{i}_{L2}}{dt}=\frac{{V}_{PV}+{v}_{C1}}{{L}_{2}}$$

The dynamics for this mode is given by

33 $$\dot{{x}_{ON}}=\left[\begin{array}{c}\begin{array}{c}\frac{{V}_{PV}+{v}_{C2}}{{L}_{1}}\\ \frac{{V}_{PV}+{v}_{C1}}{{L}_{2}}\\ -\frac{{i}_{L2}}{{C}_{1}}\end{array}\\ -\frac{{i}_{L1}}{{C}_{2}}\\ 0\\ -\frac{{v}_{C0}}{{R}_{0}{C}_{0}}\end{array}\right]$$

During mode 2,

34 $$\frac{d{i}_{L1}}{dt}=\frac{{V}_{PV}-{v}_{C2}-{v}_{C3}}{{L}_{1}}$$

35 $$\frac{d{i}_{L2}}{dt}=\frac{-{v}_{C3}}{{L}_{2}}$$

The dynamics for this mode is given by,

36 $${\dot{x}}_{OFF}=\left[\begin{array}{c}\begin{array}{c}\frac{{V}_{PV}-{v}_{C2}-{v}_{C3}}{{L}_{1}}\\ \frac{-{v}_{C3}}{{L}_{2}}\\ \frac{{i}_{L2}}{{C}_{1}}\end{array}\\ \frac{{i}_{L1}}{{C}_{2}}\\ \frac{{i}_{L1}+{i}_{L2}-{i}_{C0}}{{C}_{3}}\\ \frac{{i}_{C3}-{v}_{C0}/{R}_{0}}{{C}_{0}}\end{array}\right]$$

Here, ${i}_{C3}=\frac{d{v}_{C3}}{dt}, {i}_{C0}=\frac{d{v}_{C0}}{dt}$. Using state-space averaging over one switching period with duty cycle D, the averaged model is,

37 $$\dot{x}=D. {\dot{x}}_{ON}+\left(1-D\right).{\dot{x}}_{OFF}$$

Hence, each state derivative becomes,

38 $$\frac{d{i}_{L1}}{dt}=D. \frac{{V}_{PV}+{v}_{C2}}{{L}_{1}}+\left(1-D\right).\frac{{V}_{PV}-{v}_{C2}-{v}_{C3}}{{L}_{1}}$$

39 $$\frac{d{i}_{L2}}{dt}=D. \frac{{V}_{PV}+{v}_{C1}}{{L}_{2}}+\left(1-D\right).\frac{-{v}_{C3}}{{L}_{2}}$$

40 $$\frac{d{v}_{C1}}{dt}=D.-\frac{{i}_{L2}}{{C}_{1}}+\left(1-D\right).\frac{{i}_{L2}}{{C}_{1}}$$

41 $$\frac{d{v}_{C2}}{dt}=D.-\frac{{i}_{L1}}{{C}_{2}}+\left(1-D\right).\frac{{i}_{L1}}{{C}_{2}}$$

42 $$\frac{d{v}_{C3}}{dt}=(1-D).\frac{{i}_{L1}+{i}_{L2}-{i}_{C0}}{{C}_{3}}$$

43 $$\frac{d{v}_{C1}}{dt}=D.-\frac{{v}_{C0}}{{R}_{0}{C}_{0}}+\left(1-D\right).\frac{{i}_{C3}-{v}_{C0}/{R}_{0}}{{C}_{0}}$$

Let the state vector be,

44 $$\dot{x}=\left[\begin{array}{c}\begin{array}{c}{i}_{L1}\\ {i}_{L2}\\ {v}_{C1}\end{array}\\ {v}_{C2}\\ {v}_{C3}\\ {v}_{C0}\end{array}\right]$$

The averaged model becomes, $\dot{x}=A\left(D\right).x+B\left(D\right).{V}_{PV}$ (45).

Here, $A\left(D\right), B\left(D\right)$ are nonlinear functions of duty cycle $D$.

### Design specifications

Considering inductor design, assuming ${V}_{C}\approx {V}_{PV}$ in Eq. (24), $\Delta {i}_{L}=\frac{2{V}_{PV}\cdot D\cdot {T}_{S}}{L}$

46 $$\text{Solving for }L, {L}_{1}={L}_{2}=\frac{2{V}_{PV}\cdot D}{\Delta {i}_{L}. {f}_{s}}=\frac{2\times 500\times 0.5}{50\times {10\times 10}^{3}}=1\times {10}^{-3}H=1m$$

Here, $\Delta {i}_{L}$-inductor ripple, ${f}_{s}$-switching frequency

Considering capacitor design, solving Eq. (28) for capacitance,

47 $$C, {C}_{1}={C}_{2}={C}_{3}=\frac{{I}_{C}\cdot D}{\Delta {V}_{C}. {f}_{s}}=\frac{12\times 0.5}{6\times {10\times 10}^{3}}={100\times 10}^{-6}=100\mu F$$

Here, $\Delta {V}_{C}$-ripple voltage, ${I}_{C}$-average current through capacitor

Output capacitor

48 $${C}_{0}=\frac{{I}_{out}\cdot D}{\Delta {V}_{0}. {f}_{s}}=\frac{24\times 0.5}{12\times {10\times 10}^{3}}=100\mu F$$

In high-power-density applications, there exists a fundamental trade-off between achieving fast dynamic response and the selection of inductance values in DC–DC converter design. A lower inductance enables quicker transient response and faster tracking of sudden changes in input or load conditions, which is crucial for maintaining voltage regulation under rapidly varying solar irradiance or load fluctuations. However, this comes at the cost of increased ripple currents, which can lead to higher electromagnetic interference (EMI), increased thermal stress and potential degradation of converter components over time. On the other hand, using higher inductance reduces ripple and improves current smoothing, enhancing component reliability and efficiency, but it also slows down the converter’s dynamic response and increases size, weight, and cost, which are critical constraints in compact, high-density designs. Therefore, in the context of the proposed Active X^2^G Boost converter, optimal inductance selection must balance these competing objectives ensuring sufficiently fast response to dynamic conditions without compromising system efficiency or violating space and thermal constraints. Reducing the inductance to 1mH increases the ripple current to 50A for the same operating condition. However, lower inductance simultaneously improves response time, since smaller inductors allow the current to change more rapidly in response to control actions, reducing the time constant of the converter.

## Mathematical modelling of proposed hybrid Siberian Tiger-Meerkat optimized PI controller

In this section, PI controller gain parameters are optimized using the proposed HSTM algorithm, which is a population based stochastic optimization technique inspired by the natural behaviours of Siberian Tigers and Meerkats. This algorithm integrates MOA with STO. The STO excels in local exploitation and fine-tuning near the optimal solution, while MOA offers strong global exploration capabilities to avoid premature convergence. By combining these two, the proposed HSTM-PI controller achieves a balanced optimization process that accelerates convergence while maintaining solution accuracy and stability. Compared to single-algorithm approaches which may either converge slowly or risk local optima entrapment, the HSTM hybrid enhances both speed and precision of control tuning. While other hybrid algorithms exist, many introduce significant computational overhead or require complex parameter tuning. In contrast, HSTM maintains a lower computational burden and simpler structure, making it more practical for real-time embedded applications such as FPGA based control in EV charging systems. The Meerkat optimization behaviour and Siberian Tiger optimization is discussed in the following sub-sections.

### Meerkat optimization behaviour

In the HSTM algorithm, the initial phase focuses on exploration using MOA principles. Each solution candidate, representing a potential $\left[{K}_{p},{K}_{i}\right]$ pair is modelled as a meerkat operating in either safe or predator-alert mode. In safe mode, each meerkat explores the search space using one of two behaviours. The first is the outward search, which mimics natural foraging through random spatial dispersion. Here, the position of a candidate at iteration $t+1$, denoted ${X}_{i}^{(t+1)}$ is updated based on a random direction vector $R$ and a decreasing step-size $s$, following the expression,

49 $${X}_{i}^{(t+1)}={X}_{i}^{(t)}+s\cdot R$$

where $R\in {\left[-\text{1,1}\right]}^{D}$ introduces randomized directionality in a D-dimensional space. The factor $s$ is decreased with iterations to allow coarse exploration at the beginning and finer adjustments as the algorithm progresses. This strategy enables widespread distribution of agents early in the search to prevent premature convergence. The second behaviour under safe mode is termed cooperative search, which reflects social interactions among meerkats. In this strategy, each meerkat adjusts its position by referencing the position of another randomly selected peer ${X}_{j}^{(t)}$, using the relation,

50 $${X}_{i}^{(t+1)}={X}_{i}^{(t)}+\lambda \left({X}_{j}^{(t)}-{X}_{i}^{(t)}\right)$$

Here, $\lambda \in \left[\text{0,1}\right]$ is a scalar weight that determines the degree to which an agent is influenced by its peer. This update rule facilitates convergence through mutual information exchange, enabling agents to collaboratively explore promising regions of the solution space while retaining individualized behaviour. In the event that the environment is perceived as unsafe, the algorithm triggers a more reactive behavioural mode to prevent the population from stagnating or clustering around poor optima. In this predator-alert mode, two alternate behaviours are modelled to induce rapid adaptation, which are fleeing and fighting. In the fleeing strategy, each agent perturbs its current position by a large random offset, mimicking the emergency scattering of meerkats in response to danger. This is mathematically defined as,

51 $${X}_{i}^{(t+1)}={X}_{i}^{(t)}+{\Delta }_{rand}$$

where, ${\Delta }_{rand}$ is a randomly generated perturbation vector with relatively high variance, thereby encouraging abrupt and wide-ranging movements away from the current local region. This mechanism serves to reintroduce diversity into the population and mitigate the risk of local minima entrapment. Alternatively, in the fighting behaviour, the agents converge toward the best solution found so far, reflecting coordinated group defence. This convergence is modelled by,

52 $${X}_{i}^{(t+1)}={X}_{i}^{(t)}+\mu \left({X}_{best}^{(t)}-{X}_{i}^{(t)}\right)$$

**Fig. 7:**
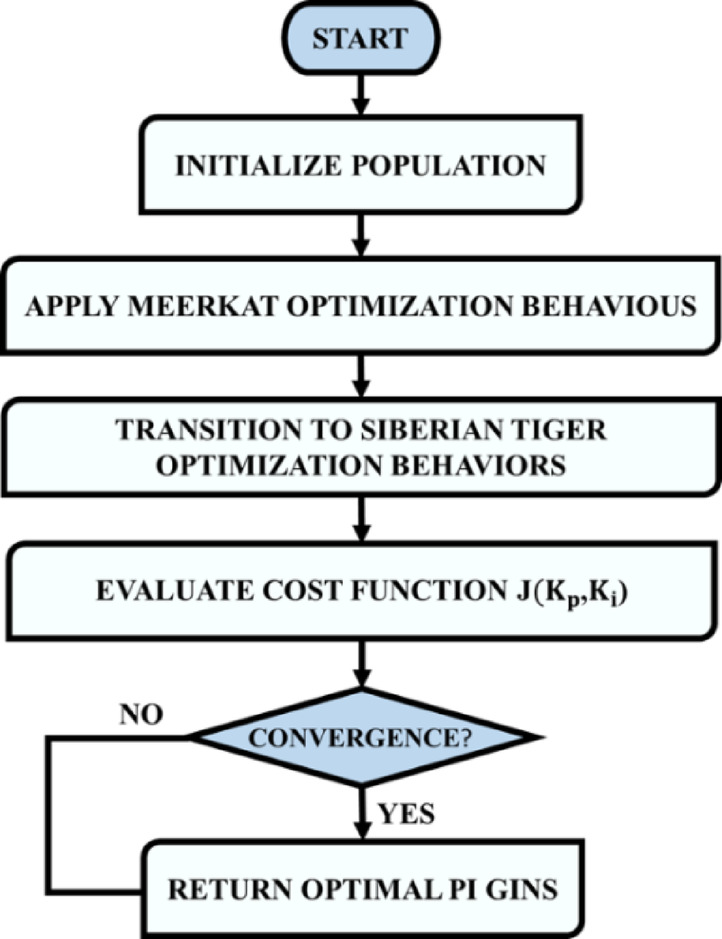
Workflow of the proposed HSTM optimized PI controller.

In this expression, ${X}_{best}^{(t)}$ denotes the globally best-performing candidate at iteration $t$ and $\mu \in \left[\text{0,1}\right]$ is a convergence coefficient that modulates the rate at which the agent is drawn toward the best-known position. This exploratory phase allows wide sampling of the search space to identify potentially optimal gain regions and workflow of the proposed HSTM optimized PI controller is shown in Fig. 7.

### Siberian Tiger optimization behaviour

Once the exploratory phase guided by Meerkat Optimization concludes, the HSTM algorithm transitions into an intensive exploitation stage governed by STO dynamics. In this phase, each candidate solution engages in a structured search process composed of two key behaviours, which are the prey hunting phase and the bear fighting phase. These behaviours enable the algorithm to refine previously explored solutions and accelerate convergence toward an optimal or near-optimal controller configuration. During the prey hunting phase, each tiger initially identifies a better-performing solution from the population to serve as its prey and subsequently attempts to move towards it. The prey attack step aims to propel the tiger aggressively in the direction of the selected prey. The updated position of the tiger at iteration $t+1$ is expressed as,

53 $${X}_{i}^{(t+1)}={X}_{i}^{(t)}+\alpha \cdot\Phi \cdot \left({X}_{p}^{\left(t\right)}-{X}_{i}^{\left(t\right)}\right)$$

In this equation,${X}_{i}^{(t)}$ is the current position of the tiger, $\alpha \in \left[\text{0,1}\right]$ is a randomly selected scaling factor that controls the step size and $\Phi \in \left\{-1,+1\right\}$ is a stochastic direction selector. The presence of $\Phi$ enables the algorithm to either pursue or slightly deviate from the prey’s path, enhancing search diversity and preventing premature convergence around suboptimal candidates. After the initial attack, the tiger refines its trajectory through the chase step, which performs a localized search around the prey’s last known position. This phase focuses on exploiting the neighbourhood of a promising solution and is modelled as,

54 $${X}_{i}^{(t+1)}={X}_{p}^{attack}+\beta \cdot \left(U-L\right)\cdot \left(1-\frac{t}{T}\right)\cdot R$$

Here, ${X}_{p}^{attack}$ is the previous prey position, $U$ and $L$ are upper and lower bounds and $T$ is the maximum iteration count. In addition to the prey hunting mechanism, each tiger also undergoes a bear fighting phase, which serves as an auxiliary intensification process by introducing competitive interactions between candidate solutions. In this phase, the tiger identifies a randomly chosen peer from the population to act as a bear opponent and performs an attack step. The resulting position update is governed by,

55 $$X_{i}^{{(t + 1)}} = X_{i}^{{(t)}} + \gamma \cdot \Phi ^{\prime } \cdot \left( {X_{k}^{{\left( t \right)}} - X_{i}^{{\left( t \right)}} } \right)$$

where, ${X}_{i}^{(t)}$ is the position of a randomly selected peer, $\gamma \in \left[\text{0,1}\right]$ is a random step size controlling the extent of the movement and ${\Phi }{\prime}\in \left\{-1,+1\right\}$ is a sign factor that allows the tiger to either approach or diverge from the bear’s position. This stochastic movement facilitates additional exploration within the current local basin, promoting solution refinement from a different perspective than that offered by the prey-based movements. The conflict step provides further localized perturbation, allowing for subtle adjustments to the tiger’s position to fine-tune its performance. This step is mathematically represented as,

56 $${X}_{i}^{(t+1)}={X}_{i}^{(t)}+\delta \cdot \mathcal{N}\left(\text{0,1}\right)$$

where, $\delta$ is a small scalar constant and $\mathcal{N}\left(\text{0,1}\right)$ denotes a standard normal distribution used to generate a Gaussian random vector. This operation introduces a controlled, zero-mean disturbance that simulates fine motor movements, emulating the tactical adjustments made by a tiger during close-quarters combat. It is particularly effective in preventing convergence stagnation by continually injecting micro-variations into the search trajectory.

### Optimization phases and control loop integration of HSTM optimized PI controller

The HSTM algorithm operates in two sequential phases to optimize PI controller gains for converter voltage regulation. The first phase, from iteration 1 to ${T}_{E}$, uses Meerkat Optimization to explore the search space, promoting diversity and avoiding local optima through random diffusion and cooperative behaviours. The second phase, from ${T}_{E}+1$ to ${T}_{max}$, applies STO to exploit the most promising regions using prey-chasing and fine-grained local refinements. Each candidate solution is a gain vector ${X}_{i}=\left[{K}_{p,i},{K}_{i,i}\right]$. Its performance is evaluated using a fitness function $J\left({K}_{p},{K}_{i}\right)$, which measures output voltage error over a simulation period $T$. The objective function for evaluation is defined as,

57 $$J\left({K}_{p},{K}_{i}\right)={\int }_{0}^{T}{\left({V}_{ref}-{V}_{out}(t)\right)}^{2}dt$$

The algorithm iteratively updates the population using the described stochastic dynamics, retaining, and refining the solution with the lowest cost $J$. Upon convergence, the optimal gains $\left[{K}_{p}^{*},{K}_{i}^{*}\right]$ are selected and applied to the converter’s feedback control loop. The resulting HSTM-PI controller achieves superior regulation accuracy and dynamic response, attributed to the hybrid algorithm’s ability to navigate complex search landscapes efficiently. The hybridization of MOA and STO overcomes critical limitations inherent to each method. While MOA provides robust exploration, it tends to converge slowly and lacks precision near the optimal solution. Conversely, STO offers rapid convergence through aggressive exploitation but becomes trapped in local optima due to limited diversity. By merging these algorithms, HSTM capitalizes on the exploratory breadth of MOA and the exploitative sharpness of STO. This combination results in faster convergence, avoidance of premature stagnation and improved robustness in tuning PI controllers for nonlinear converter dynamics. The process followed to implement the HSTM optimized PI controller is demonstrated in Algorithm 1.

**Algorithm 1:**
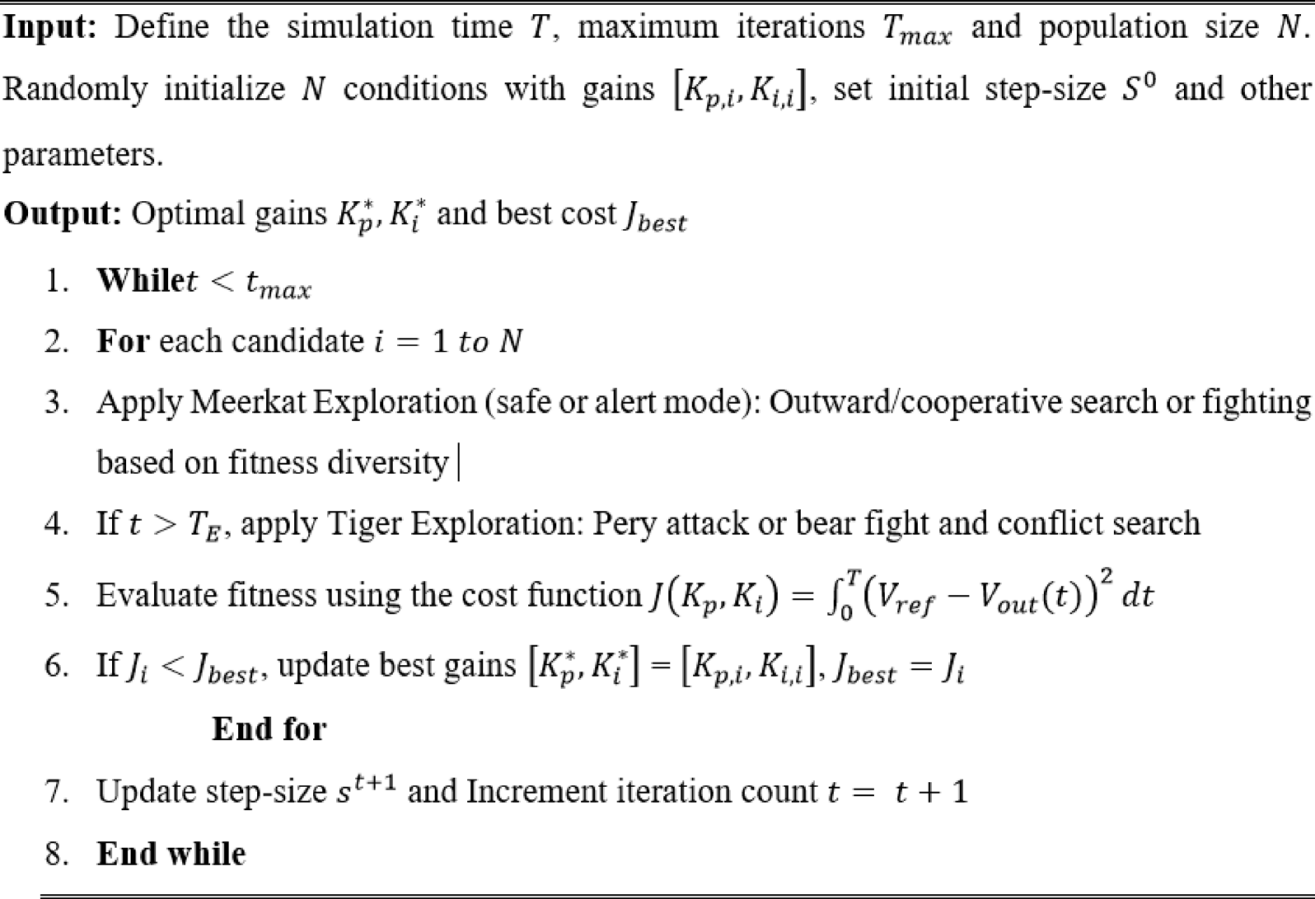
HSTM optimized PI controller for Active X^2^G Boost converter.

The hybrid optimization framework enables adaptive tuning of PI gains even as system dynamics change due to aging components or thermal variations. The controller’s adaptive nature allows it to compensate for parameter drift by recalibrating control responses in real time, thus maintaining voltage regulation and system stability. Moreover, the HSTM-PI design includes noise-tolerant feedback filtering mechanisms that mitigate the effects of sensor inaccuracies, ensuring smooth control signals and preventing instability. In grid-tied operations, where communication delays can affect synchronization and control timing, the controller’s fast convergence and predictive tuning behavior helps maintain robust performance despite transient delays.

## Modelling of bidirectional converter with battery

**Fig. 8:**
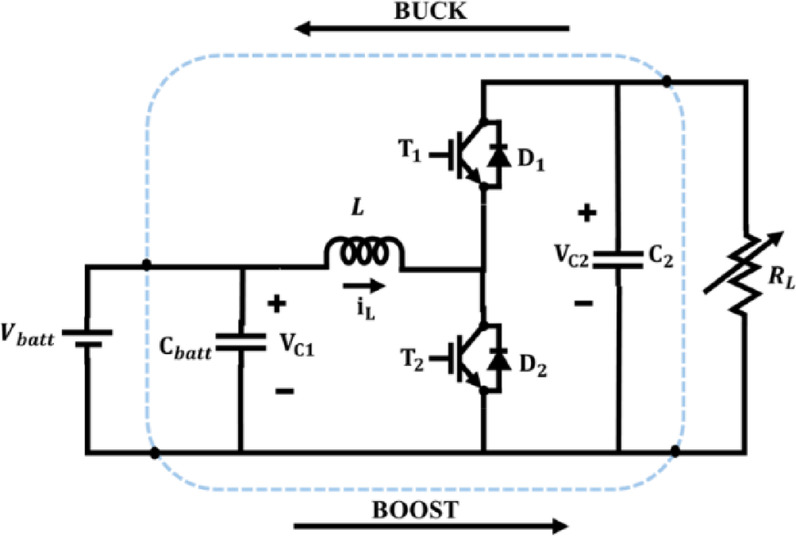
Bidirectional DC–DC converter.

The bidirectional converter as illustrated in Fig. 8 is employed in this system for facilitating controlled energy flow between DC bus and battery system. From the structure of bidirectional DC–DC converter, terms ${V}_{batt}$ refers to battery voltage, ${V}_{dc}$ refers to DC bus voltage, $L$ refers to converter inductor, ${C}_{batt}$ refers to battery-side filter capacitor, ${i}_{L}$ refers to inductor current, ${i}_{batt}$ refers to battery current, $D$ is the duty ratio and ${R}_{L}$ refers to load resistance. The converter can be configured to work either in Boost mode or in Buck mode operation.

### Boost mode operation

**Fig. 9:**
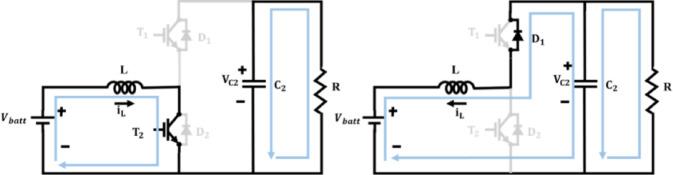
Bidirectional converter during Boost mode.

When the battery discharges to supply power to the DC bus, the converter operates in boost mode as in Fig. 9. In this case, energy flows from the low-voltage battery side to the high-voltage DC bus. Inductor stores energy during the switch ON state in Boost mode,

58 $$\frac{{di}_{L}}{dt}=\frac{{V}_{batt}-{R}_{batt}{i}_{L}}{L}$$

59 $$\frac{{dv}_{batt}}{dt}=\frac{{i}_{batt}-{i}_{L}}{{C}_{batt}}$$

Inductor discharges into the DC bus during switch OFF state in Boost mode,

60 $$\frac{{di}_{L}}{dt}=\frac{{V}_{batt}-{V}_{dc}-{R}_{batt}{i}_{L}}{L}$$

61 $${V}_{dc}=\frac{{V}_{batt}}{1-D}$$

62 $${i}_{batt}=\frac{{I}_{dc}\left(1-D\right)}{\eta }$$

where $\eta$ is converter efficiency.

### Buck mode operation

**Fig. 10:**
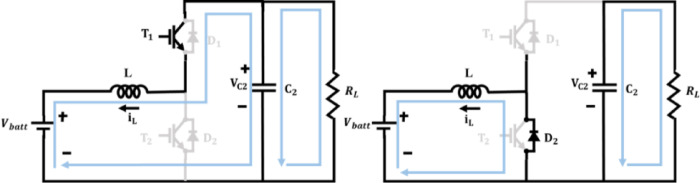
Bidirectional converter during Buck mode.

PV energy charges the battery through the converter as shown in Fig. 10. In Buck mode, when the switch is ON, energy flows from the DC bus into the inductor

63 $$\frac{{di}_{L}}{dt}=\frac{{V}_{dc}-{R}_{batt}{i}_{L}-{V}_{batt}}{L}$$

64 $$\frac{{dv}_{batt}}{dt}=\frac{{i}_{L}-{i}_{batt}}{{C}_{batt}}$$

Inductor discharges into the battery via freewheeling diode during switch OFF state in Buck mode,

65 $$\frac{{di}_{L}}{dt}=\frac{-{V}_{batt}-{R}_{batt}{i}_{L}}{L}$$

66 $${V}_{batt}=D\cdot {V}_{dc}$$

67 $${i}_{L}=\frac{{I}_{batt}}{D\cdot \eta }$$

On linearizing the converter using small-signal perturbations, let,

68 $${V}_{batt}={\overline{V} }_{batt}+{\widehat{v}}_{batt}, {i}_{L}={\overline{i} }_{L}+{\widehat{i}}_{L}, D=\overline{D }+\widehat{d}$$

The small-signal state-space model becomes,

69 $$\frac{d}{dt}\left[\begin{array}{c}{\widehat{i}}_{L}\\ {\widehat{v}}_{batt}\end{array}\right]=\left[\begin{array}{cc}-\frac{{R}_{batt}}{L}& -\frac{1}{L}\\ \frac{1}{{C}_{batt}}& 0\end{array}\right]\left[\begin{array}{c}{\widehat{i}}_{L}\\ {\widehat{v}}_{batt}\end{array}\right]+\left[\begin{array}{c}\frac{{V}_{dc}}{L}\\ 0\end{array}\right]\widehat{d}$$

By accounting for component-level dynamics and bidirectional operation, the proposed model enables precise control and optimal energy utilization across operational modes.

### Grid voltage synchronization

**Fig. 11:**
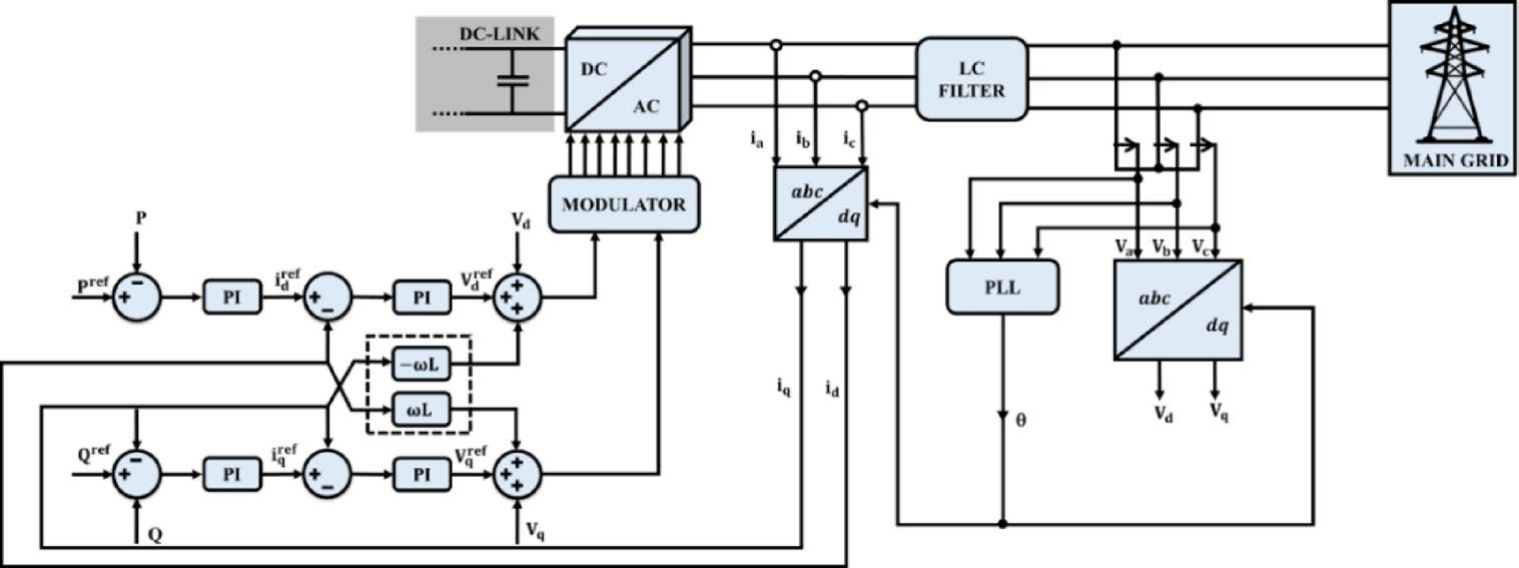
DQ theory based grid voltage synchronization.

To ensure accurate synchronization between a grid-connected inverter and the utility grid, DQ control strategy based on the transformation of three-phase quantities into the rotating $dq$ reference frameis employed. This approach decouples the control of active powerand reactive powerusing two independent PI controllers as depicted in Fig. 11. The overall control structure relies on three key elements, which are Park transformation, current control via PI regulation and Phase Lock Loop (PLL) for accurate phase angle, frequency tracking. The first step involves transforming three-phase voltages and currents from the stationary $abc$ frame to the synchronous rotating $dq$ frame using Park’s transformation. This yields two orthogonal components that rotate at the grid frequency $\omega$.

70 $$\left[\begin{array}{c}{V}_{d}\\ {V}_{q}\\ {V}_{0}\end{array}\right]=\frac{2}{3}\left[\begin{array}{ccc}\text{cos}\theta & \text{cos}\left(\theta -\frac{2\pi }{3}\right)& \text{cos}\left(\theta +\frac{2\pi }{3}\right)\\ \text{sin}\theta & \text{sin}\left(\theta -\frac{2\pi }{3}\right)& \text{sin}\left(\theta +\frac{2\pi }{3}\right)\\ \frac{1}{2}& \frac{1}{2}& \frac{1}{2}\end{array}\right]\left[\begin{array}{c}{V}_{a}\\ {V}_{b}\\ {V}_{c}\end{array}\right]$$

Here, $\theta$ is the grid angle estimated using the PLL. The same transformation is applied to the currents $\left[{i}_{a},{i}_{b},{i}_{c}\right]$ to obtain ${i}_{d}$ and ${i}_{q}$, which are used for power regulation. Filter circuit between the inverter and the grid plays a vital role in smoothing current harmonics and limiting high frequency switching components. The inductor voltage dynamics in the synchronous $dq$ frame are given by,

71 $$\frac{{di}_{d}}{dt}=\frac{1}{L}\left({V}_{d}^{inv}-{V}_{d}\right)+{\omega i}_{q}$$

72 $$\frac{{di}_{q}}{dt}=\frac{1}{L}\left({V}_{q}^{inv}-{V}_{q}\right)+{\omega i}_{d}$$

These equations describe the influence of inverter reference voltages ${V}_{d}^{inv}$ and ${V}_{q}^{inv}$ on the output grid current. The terms ${\omega i}_{q}$ and ${\omega i}_{d}$ represent cross-coupling effects between the two axes due to the rotating frame. The instantaneous active and reactive powers delivered to the grid are calculated in the dq frame using,

73 $$P=\frac{3}{2}\left({V}_{d}{i}_{d}+{V}_{q}{i}_{q}\right) Q=\frac{3}{2}\left({V}_{q}{i}_{d}+{V}_{d}{i}_{q}\right)$$

These equations are essential for generating reference currents ${i}_{d}^{ref}$ and ${i}_{q}^{ref}$. Under ideal conditions, ${i}_{d}$ regulates real power and ${i}_{q}$ regulates reactive power independently. To ensure accurate power injection into the grid, reference values of current are derived.

74 $${i}_{d}^{ref}=\frac{2}{3}\cdot \frac{{P}^{ref}}{{V}_{d}}, {i}_{q}^{ref}=-\frac{2}{3}\cdot \frac{{Q}^{ref}}{{V}_{d}}$$

These current references serve as the input to the current controller. To track ${i}_{d}^{ref}$ and ${i}_{q}^{ref}$, PI controllers are used along with cross-coupling compensation to decouple the *d* and *q* channels.

75 $${V}_{d}^{ref}=PI\left({i}_{d}^{ref}-{i}_{d}\right)+\omega {Li}_{q}$$

76 $${V}_{q}^{ref}=PI\left({i}_{q}^{ref}-{i}_{q}\right)+\omega {Li}_{d}$$

Here, the PI controller minimizes the error between actual and reference current, while the feedforward terms $\omega {Li}_{q}$ and $\omega {Li}_{d}$ cancel the coupling effect introduced by the rotating *dq* frame. The computed ${V}_{d}^{ref}$ and ${V}_{q}^{ref}$ values are transformed back into the three-phase frame via inverse Park transformation,

77 $$\left[\begin{array}{c}{V}_{a}^{ref}\\ {V}_{b}^{ref}\\ {V}_{c}^{ref}\end{array}\right]={T}_{dq}^{-1}\left(\theta \right)\left[\begin{array}{c}{V}_{d}^{ref}\\ {V}_{q}^{ref}\end{array}\right]$$

These voltages are then used by the PWM modulator to generate gating signals for the 3ϕ VSI.

The single-switch configuration of the converter inherently reduces conduction paths and power losses, making it more thermally manageable under high current loads compared to multi-switch topologies. The integration of a quasi-Z-source network ensures continuous input current and reduced ripple, which enhances component lifespan and system reliability under demanding charging conditions. Although the HSTM-PI controller introduces a hybrid metaheuristic structure, it is specifically designed for efficient convergence with minimal tuning requirements, and its implementation on FPGA platforms enables real-time operation with manageable resource utilization. Moreover, the modular nature of the proposed converter allows for parallel configuration and power sharing, making it adaptable to multi-port or multi-vehicle charging scenarios. With proper synchronization and load-balancing algorithms, the converter can support scalable energy distribution in smart EV infrastructures, thus reinforcing its practical applicability and technical merit.

## Results and discussion

To validate proposed system incorporating the Active ${X}^{2}$G Boost converter and HSTM-PI controller, a series of simulations were conducted using MATLAB. The analysis includes three distinct test scenarios to evaluate system reliability, voltage stability, and dynamic response under various real-world operating conditions. In Case 1, the system is tested under constant irradiance and temperature to examine its steady-state behaviour. Case 2 investigates the converter’s adaptability to dynamic environmental conditions with fluctuating irradiance.

In Case 3, the PV source is deliberately disconnected at 0.35 s to assess the bidirectional converter and battery storage system to maintain uninterrupted power delivery. System parameters utilized to implement the Active ${X}^{2}$G Boost converter and the HSTM-PI controller are listed in Table 1.

**Table 1:** System specifications.

Attributes	Values	Attributes	Values
PV system			
Maximum power of module	175.09W	Voltage at Maximum Power	36.6 V
Open Circuit Voltage	43.99 V	Short-Circuit Current	5.17A
Current at Maximum Power	4.78 A	Number of Parallel Strings	120
Diode Ideality factor	0.988	Series modules per string	9
${X}^{2}$GBoost converter			
${L}_{1},{L}_{2}$	1 mH	${C}_{1},{C}_{2},{C}_{3},{C}_{0}$	$100\mu F$
Switching frequency	10 kHz	Duty cycle	0.5
Battery			
No. of battery units	20	Battery type	Lithium-Ion
Ratings	150 Ah, 12 V	Depth of discharge	80%
HSTM optimized PI controller gains			
${k}_{p}=0.04$ and ${k}_{i}=0.02$			

### Case 1: Performance evaluation under steady-state PV conditions

**Fig. 12:**
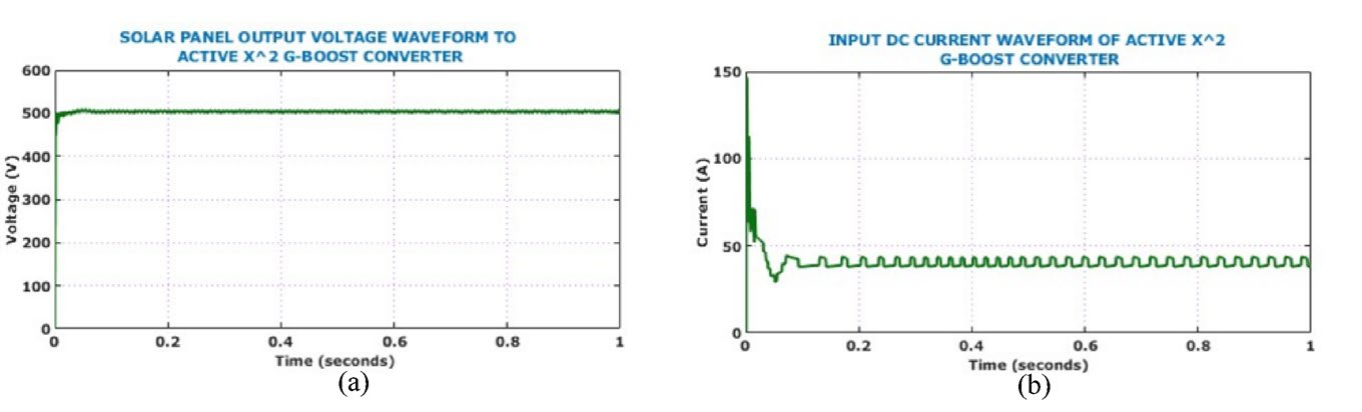
Input electrical behaviour of the converter under constant solar conditions: (**a**) voltage, (**b**) current.

**Fig. 13:**
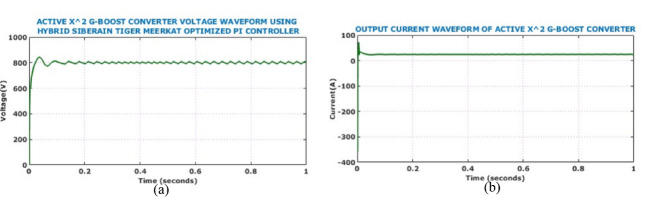
Output electrical behaviour of the converter under constant solar conditions (**a**) voltage (**b**) current.

In case 1, the intensity is maintained stable at $950 \text{W}/{\text{m}}^{2}$, while the temperature is maintained stable at $34^\circ{\rm C}$. Figure 12 shows input side waveforms of Active ${X}^{2}$ Boost converter under constant environmental conditions. The solar panel output voltage waveform remains steady at 500 V following initial transients. Active ${X}^{2}$ Boost converter can boost thevoltage and the resultant output voltage, current of the converter is shown in Fig. 13.

**Fig. 14:**
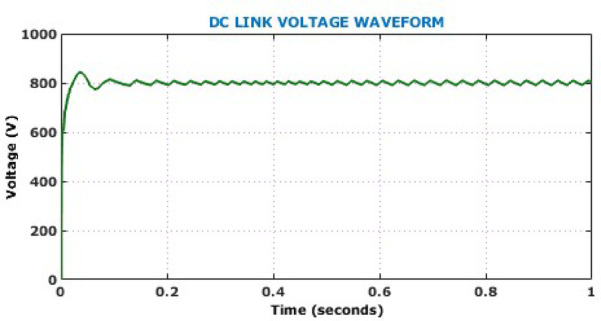
DC link voltage under constant solar levels.

**Fig. 15:**
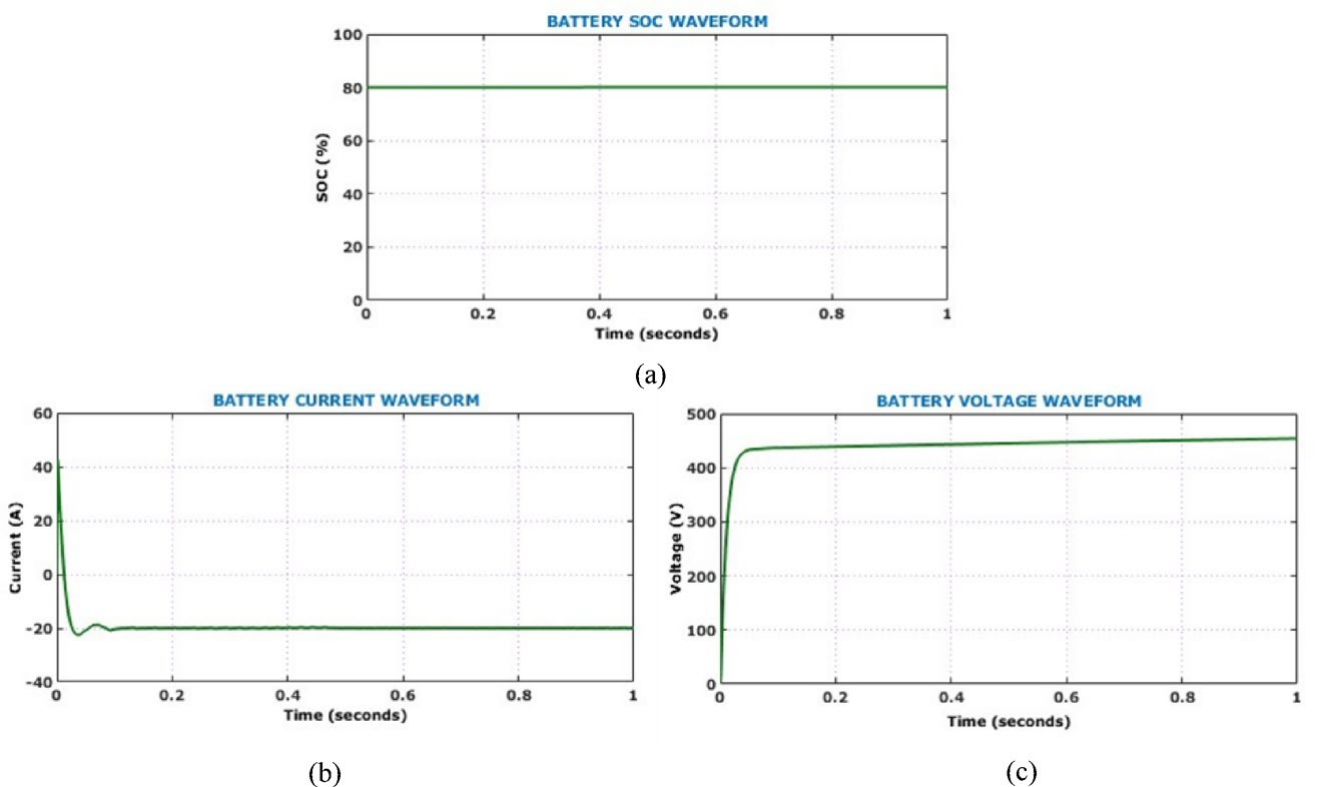
Battery performance under constant solar conditions (**a**) SoC, (**b**) current, (**c**) voltage.

Presence of minimal oscillations during the transient phase followed by a consistent voltage level confirms the controller’s effective regulation capability and rapid dynamic response. On the other hand, the output current waveform exhibits a smooth and stable profile. Absence of oscillations and distortion in the current waveform suggests excellent output filtering and efficient load handling capacity of the proposed converter. The DC link voltage waveform depicted in Fig. 14 demonstrates system capability in maintaining voltage stability under constant solar levels. The minimal steady-state ripple reflects the converter’s high efficiency and the robustness of the HSTM-PI controller in sustaining a stable DC link. This stability is crucial for reliable downstream interfacing with inverters and battery storage, confirming the proposed system’s suitability for real-time EV charging applications. Figure 15 illustrates the battery performance characteristics during the operation under constant solar conditions. The State of Charge (SoC) remains constant at 80%, while the battery voltage waveform rises sharply at the start of the simulation and stabilizes just below 480 V, confirming that the battery reaches its target voltage quickly and maintains it with minimal ripple.

### Case 2: Performance evaluation under dynamic environmental conditions

**Fig. 16:**
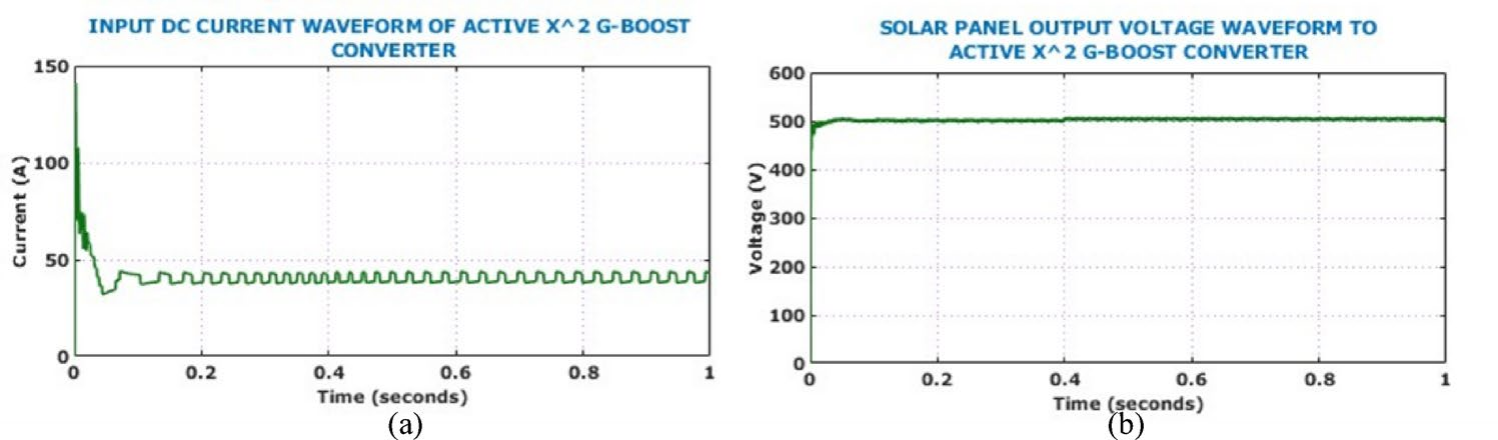
Input electrical behaviour of the converter under dynamic solar conditions (**a**) current (**b**) voltage.

In case 2, the system is ascertained for dynamic environmental conditions by varying the intensity. Accordingly, the intensity is varied to $960 \, \text{W}/{\text{m}}^{2}$ from $840 \, \text{W}/{\text{m}}^{2}$ at 0.4 s, which allows for the assessment of converter adaptability and ability of the HSTM-PI controller to maintain voltage stability and system response during abrupt environmental changes. The voltage and current waveforms shown in Fig. 16 provides the input electrical behaviour of Active ${X}^{2}$G Boost converter under dynamic solar conditions.

**Fig. 17:**
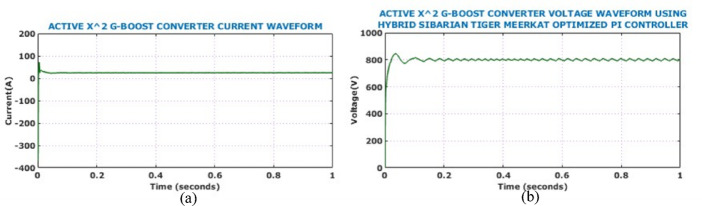
Output electrical behaviour of the converter under dynamic solar conditions (**a**) current (**b**) voltage.

**Fig. 18:**
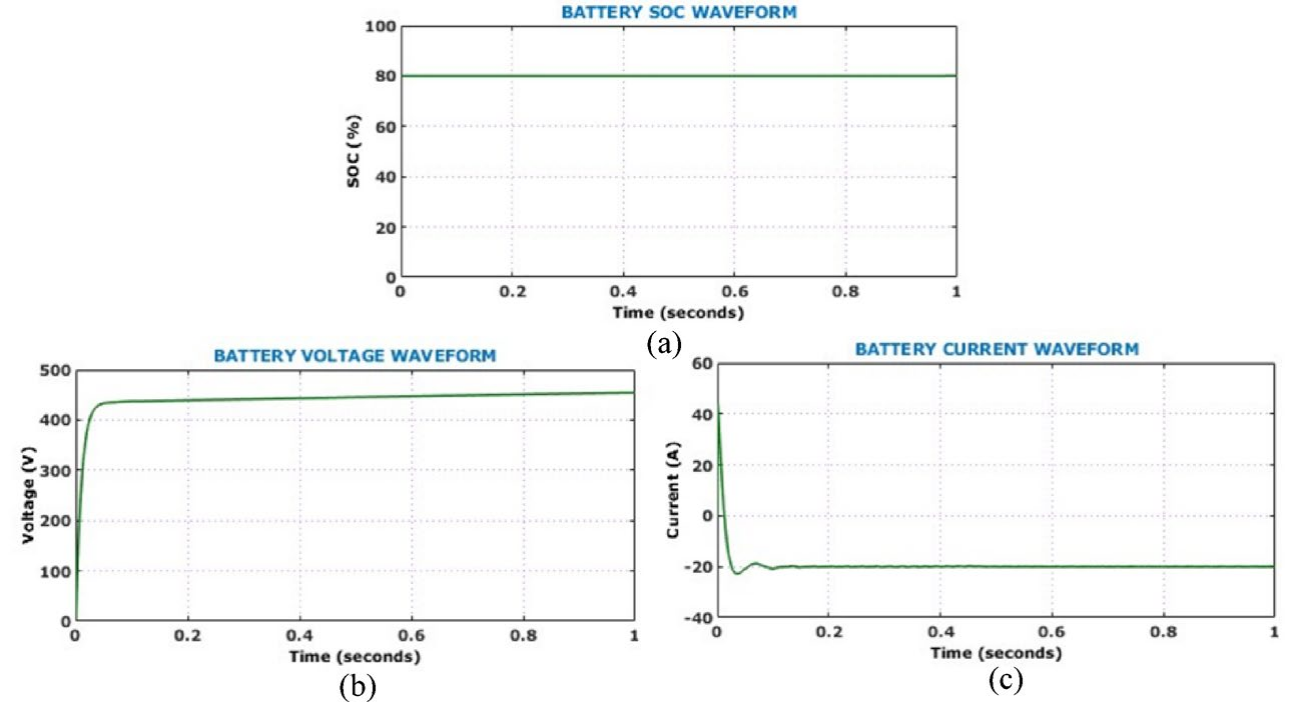
Battery performance parameters under dynamic solar conditions (**a**) SoC, (**b**) voltage, (**c**) current.

**Fig. 19:**
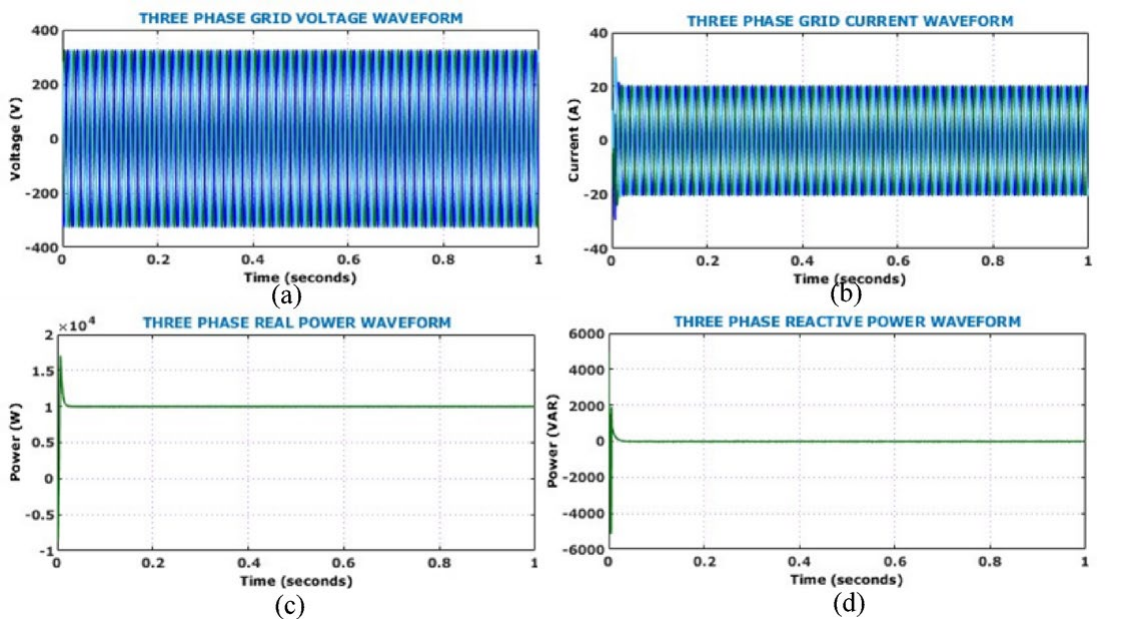
Analysis of grid-side electrical behaviour (**a**) grid voltage (**b**) grid current (**c**) real power (**d**) reactive power.

Figure 17 shows the output side voltage, current waveforms of Active X^2^ G Boost under dynamically changing solar conditions. The voltage waveform, regulated by the HSTM-PI controller, exhibits a slight increase in ripple amplitude in response to irradiance variation, but effectively stabilizes at 800 V. Therefore, the proposed controller exhibits strong voltage regulation capability despite fluctuating environmental conditions. Output current waveform remains relatively stable throughout the analysis and negligible fluctuation under dynamic solar conditions. Figure 18 illustrates the behaviour of the battery system, specifically when irradiance levels vary during the simulation. Grid-side electrical performance is shown in Fig. 19. Voltage and current waveforms are balanced and sinusoidal, indicating proper synchronization and control. The real power stabilizes just above 10 kW, confirming steady active power delivery. The reactive power quickly settles near zero, demonstrating effective reactive power compensation and power factor correction.

### Case 3: Evaluation of system reliability during PV source interruption

**Fig. 20:**
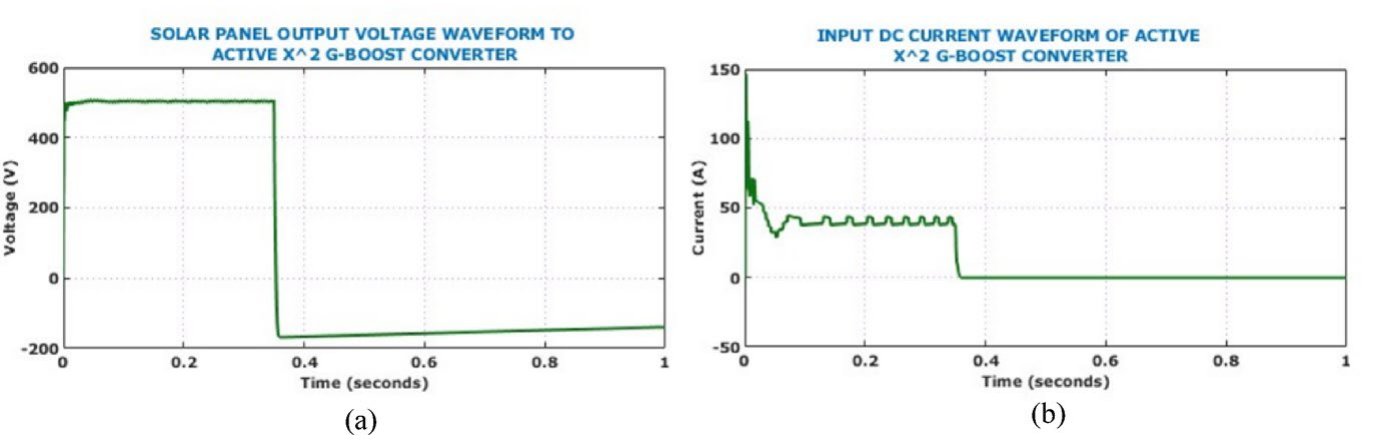
Input electrical behaviour of Active ${X}^{2}$ Boost converter during PV disconnection. (**a**) Voltage (**b**) current.

In this scenario, the PV source is deliberately disconnected at 0.35 s to evaluate the system’s ability to maintain uninterrupted power delivery using the battery backup through the bidirectional DC–DC converter. This case simulates a sudden solar outage, such as shading or fault conditions, to examine the resilience and responsiveness of the system architecture. Upon PV disconnection, the bidirectional converter transitions to boost mode to sustain DC link and load requirements. Figure 20 shows the input side waveforms of Active ${X}^{2}$ Boost converter when the PV source is intentionally disconnected at 0.35 s. Prior to disconnection, the solar panel output voltage remains stable around 500 V, and the input current shows a consistent ripple pattern, indicating usual energy transfer.

**Fig. 21:**
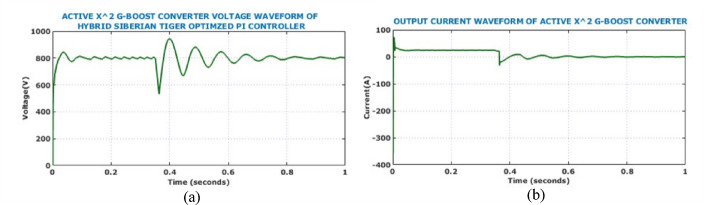
Output electrical behaviour of Active ${X}^{2}$G Boost converter during PV disconnection. (**a**) Voltage (**b**) current.

**Fig. 22:**
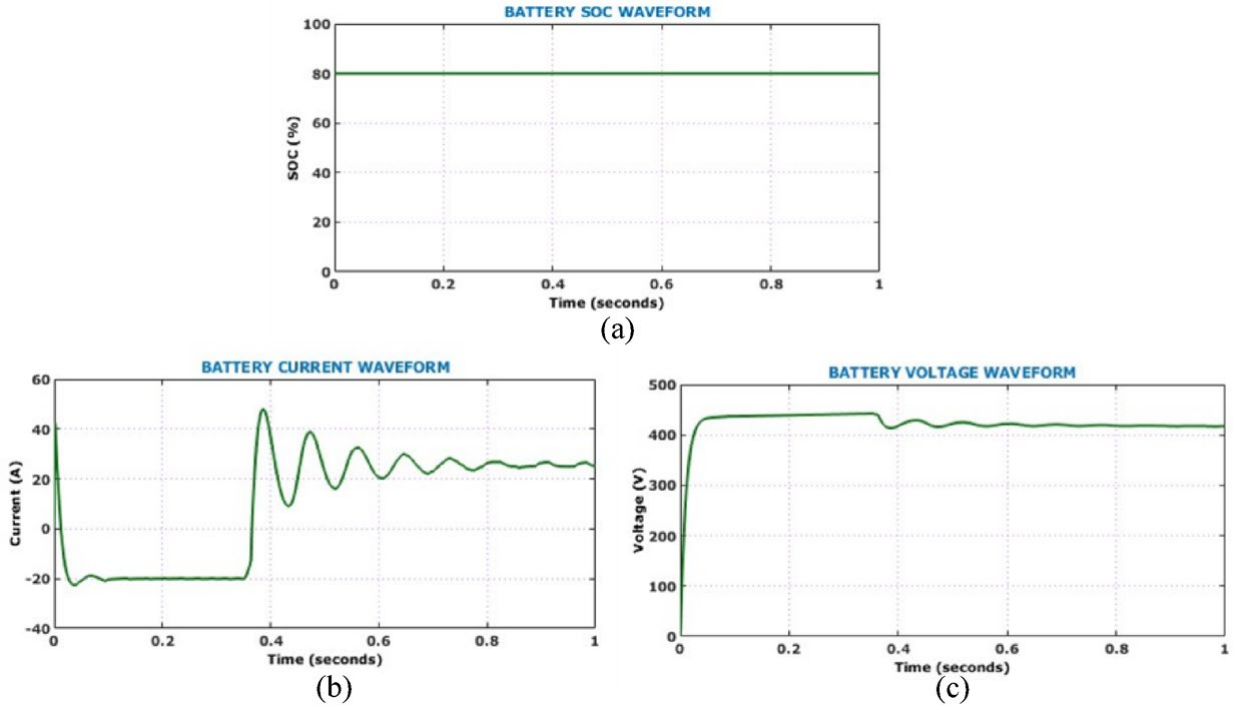
Battery performance during PV disconnection (**a**) SoC (**b**) current (**c**) voltage.

During the occurrence of PV interruption, the panel voltage drops abruptly to near zero, with a slight negative dip due to circuit transients, confirming total disconnection. Correspondingly, the converter current input falls rapidly to zero, indicating that no energy is being drawn from the PV source. Output voltage, current waveforms of the Active X^2^G Boost converter and battery behaviour during PV source disconnection at 0.35 s is presented in Figs. 21 and 22 respectively.

**Fig. 23:**
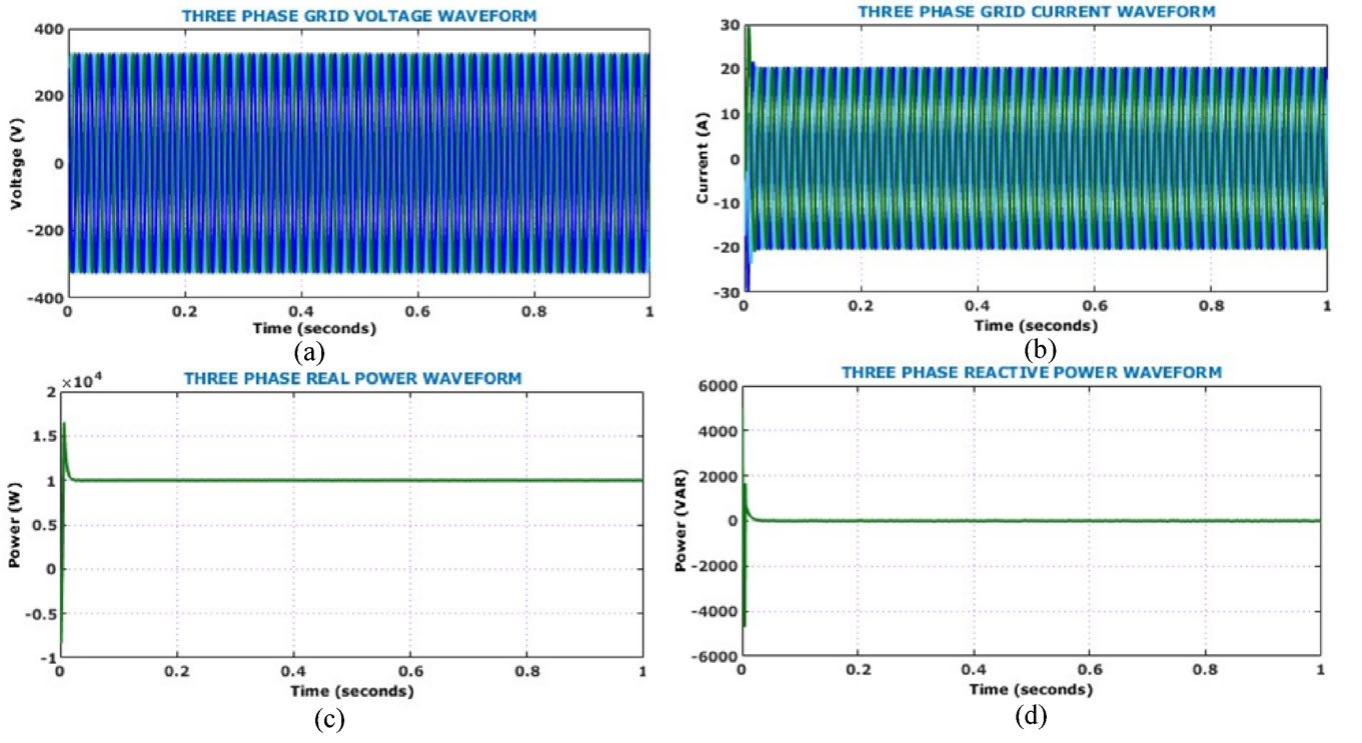
Analysis of grid-side electrical behaviour under PV disconnection (**a**) three-phase grid voltage (**b**) three-phase grid current (**c**) real power (**d**) reactive power.

**Fig. 24:**
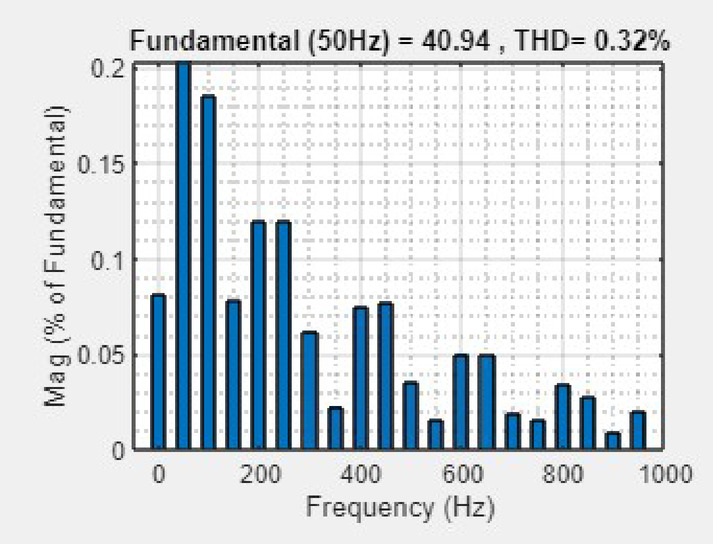
THD of the current waveform.

The SoC remains constant at approximately 80%, indicating that the battery has sufficient reserve capacity. Before the PV disconnection, the battery current is negative, indicating that it is operating in charging mode. At the point of disconnection, the current reverses direction and spikes positively, indicating a shift to discharging mode as the battery begins supplying power to support the system. After a few oscillations, the current stabilizes and ensures effective transition control. Similarly, the battery voltage shows a brief dip corresponding to the disconnection. However, it quickly stabilizes and reflect the system capability to maintain output voltage despite PV input loss. Grid side voltage, current, real power and reactive power are presented in Fig. 23. It is observed that, proposed system maintains grid stability and quality through battery support during the PV outage. Figure 24 shows the simulated THD spectrum of the system output. The observed waveform reveals a strong 50 Hz component and a low THD of 0.32%, confirming superior harmonic mitigation.

### Experimental set-up and hardware results

**Fig. 25:**
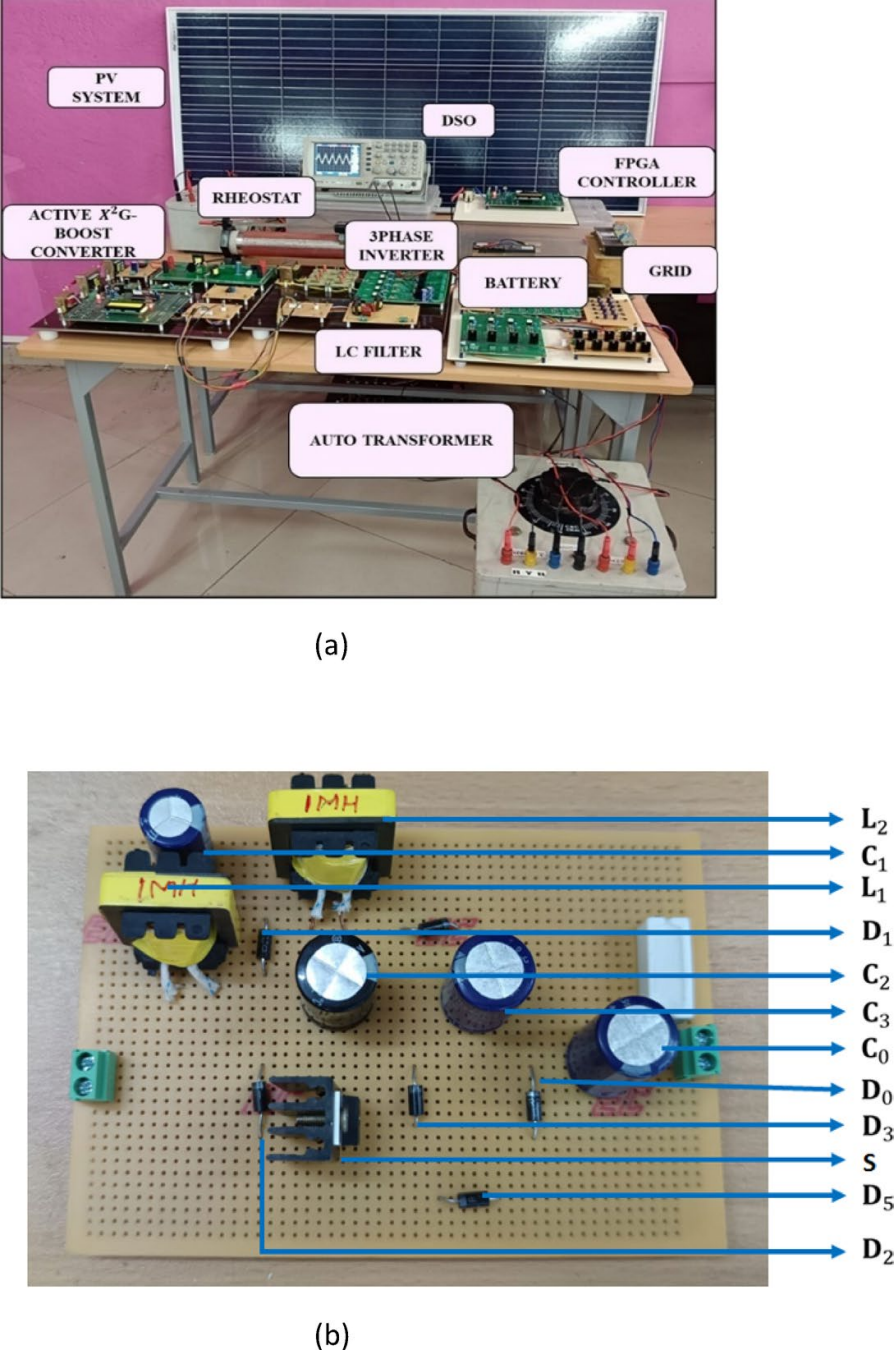
Hardware implementation of proposed Active X^2^ G Boost converter with HSTM-PI controller.

Experimental laboratory prototype implementation to validate the performance of the proposed setup is shown in Fig. 25a and the Active X^2^G Boost converter is shown in Fig. 25b. The setup includes key components such as a PV panel of 10 kW, which acts as the primary source, and a rheostat used for simulating load variations. The Active ${X}^{2}$G Boost converter steps up the PV voltage and interfaces with the battery energy storage system, while the FPGA controller manages real-time control, including PI tuning. Data acquisition and waveform analysis are carried out using a digital storage oscilloscope (DSO), capturing critical voltage and current waveforms across converter stages, battery terminals, and inverter outputs. The FPGA also facilitates logging of controller response time under varying irradiance and load conditions. The switching frequency adopted is 10 kHz and the diode used is of range IN4007-1000 V/1A. The range of battery is 120 V, 100Ah and grid is 415 V, 1 kW whereas the driver circuit used is TLP250-35 V/1.5A. The load type used is rheostat which is adjustable to simulate dynamic load conditions. The sampling rate is typically in the range of 10–100 kHz. The grid interface parameters are given by 415 V, 10mH inductive filter, 5A for ensuring smooth integration with the utility.

**Fig. 26:**
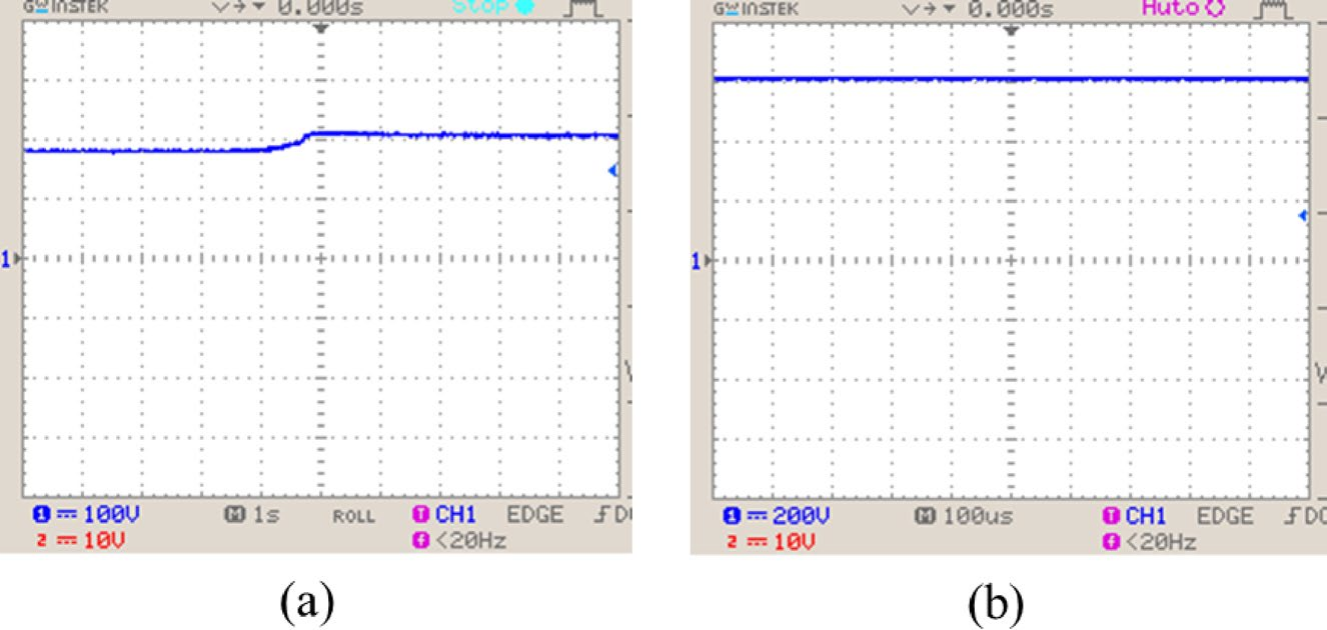
Hardware results of Active ${X}^{2}$G-Boost converter (**a**) input voltage and (**b**) output voltage.

Experimental voltage waveforms captured from the hardware setup of the Active ${X}^{2}$G Boost converter is shown in Fig. 26. Input voltage shows a smooth rise and stabilization, while the output voltage remains steady, validating the converter’s real-time performance and consistency with simulation results. These observations demonstrate reliable voltage conversion and control under practical operating conditions.

**Fig. 27:**
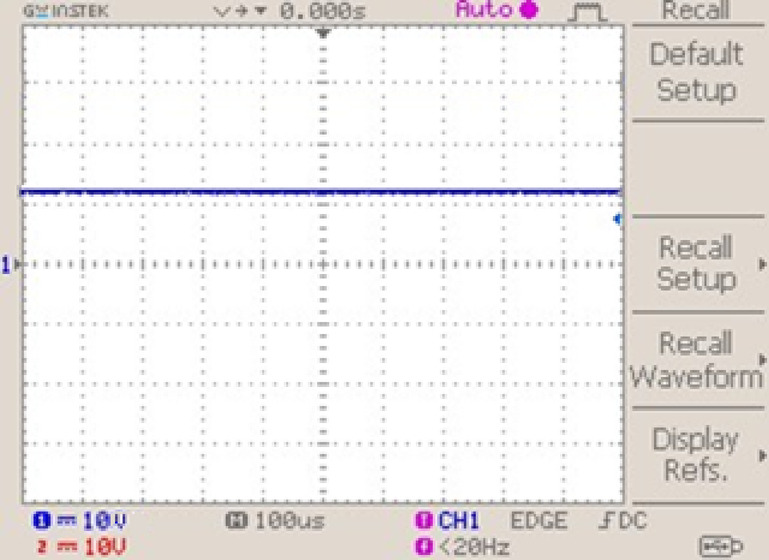
Battery voltage waveform.

Figure 27 presents the battery voltage waveform obtained from the hardware setup. The waveform remains stable over time, indicating that the battery voltage is consistently regulated without noticeable ripple or fluctuation. This steady profile confirms effective bidirectional converter control during battery charging, ensuring smooth voltage delivery and reliable system performance.

**Fig. 28:**
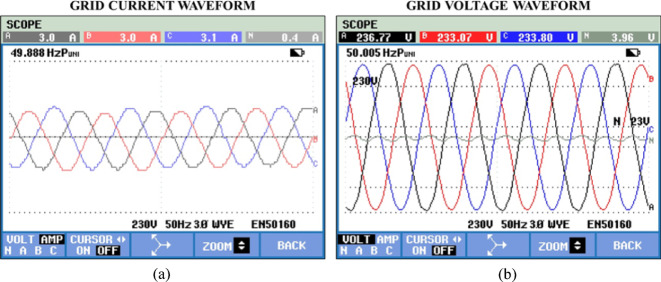
Hardware results (**a**) Grid voltage and (**b**) Grid current.

**Fig. 29:**
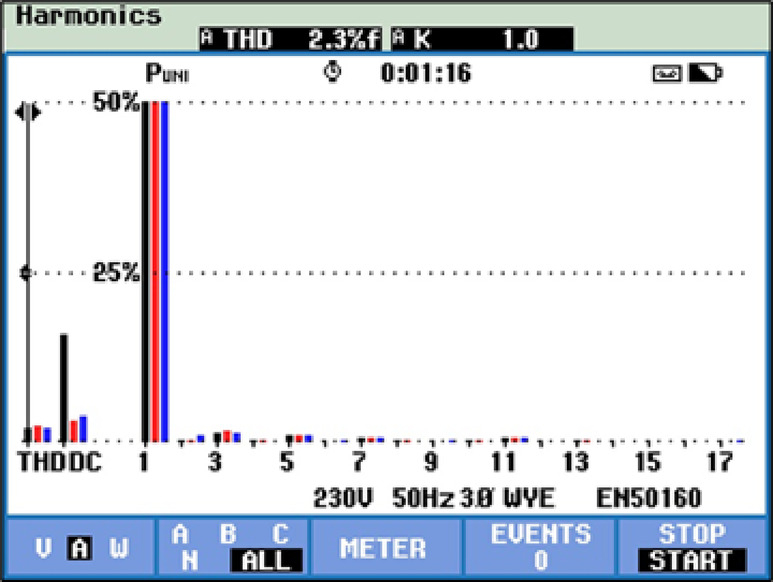
THD of the current waveform using the hardware model.

Hardware-captured waveforms of grid are shown in Fig. 28. Both signals exhibit accurate, sinusoidal nature, indicating proper synchronization and power injection into the grid. Figure 29 illustrates the measured THD of the system output using hardware, with a recorded value of 2.3%.

### Comparative performance evaluation

Comparative performance analysis presented in Table 2 highlights the performance and structural complexity of various DC–DC converters in relation to proposed Active ${X}^{2}$G Boost converter. Conventional converters exhibit moderate voltage gain, with simpler structures. However, their gain is limited and often encounter high voltage stress or control complexity. Advanced configurations such as QBC and CBC improve gain, but at the cost of increased component count and size. Other high-gain structures such as Z-Source, 3Z Network and SC/SL-SBC offer superior performance but often introduce significant design complexity, multiple inductors, and higher switching stress. In contrast, proposed converter achieves a notable voltage gain with relatively moderate component count while maintaining manageable voltage stress and continuous input current. This balance between gain enhancement and structural efficiency underscores the suitability of topology for PV-based applications requiring compact, high-performance converters with low losses.

**Table 2:** Comparative evaluation of Active ${X}^{2}$G Boost converter with prominent existing topologies.

Converter	S	C	D	I	Switch voltage	${D}_{o}$ voltage	Gain
Boost	1	1	1	1	${V}_{o}$	${V}_{o}$	$\frac{1}{1-D}$
Buck-Boost	1	1	1	1	${V}_{o}$	${V}_{o}$	$\frac{D}{1-D}$
SEPIC	1	2	1	2	${V}_{o}$	${V}_{o}$	$\frac{D}{1-D}$
Zeta	1	2	1	2	${V}_{o}$	${V}_{o}$	$\frac{D}{1-D}$
Luo	1	2	2	2	${V}_{o}$	${V}_{o}$	$\frac{1}{{\left(1-D\right)}^{2}}$
Interleaved Boost	2	2	2	2	${V}_{o}$	${V}_{o}$	$\frac{1}{1-D}$
CBC^11^	1	2	3	2	${V}_{o}$	${V}_{o}$	$\frac{1}{{\left(1-D\right)}^{2}}$
QBC^12^	1	2	3	2	${V}_{o}$	${V}_{o}$	$\frac{1}{{\left(1-D\right)}^{2}}$
Z-Source converter^15^	1	3	2	2	${V}_{o}$	${V}_{o}$	$\frac{1}{1-2D}$
SL Boost^16^	1	1	10	4	${V}_{o}$	${V}_{o}$	$\frac{1+3D}{1-D}$
3Z Network^17^	1	2	9	4	${V}_{o}$	${V}_{o}$	$\frac{{\left(1+D\right)}^{2}}{{\left(1-D\right)}^{2}}$
High Gain^18^	1	3	8	4	${V}_{o}$	${V}_{o}$	$\frac{1+D}{1-3D}$
SC/SL-SBC^19^	2	3	7	2	$\frac{{V}_{o}}{2}$	${V}_{o}$	$\frac{2-2D}{1-3D}$
Step-up Boost^23^	1	3	3	2	${V}_{o}$	${V}_{o}$	$\frac{1+{n}_{21}+{n}_{31}D}{1-D}$
Interleaved DC–DC^24^	2	3	4	2	${V}_{o}$	${V}_{o}$	$\frac{4+3n}{1-D}$
Ultrahigh DC–DC^25^	1	5	6	2	${V}_{o}$	${V}_{o}$	$\frac{1}{2+{n}_{21}+{n}_{31}}$
Proposed converter	1	4	5	2	${V}_{o}$	${V}_{PV}$	$\frac{2D}{1-D}$

The efficiency is calculated by, $\eta \left(\%\right)=\frac{{V}_{out}\times {I}_{out}}{{V}_{in}\times {I}_{in}}=\frac{800\times 24}{500\times 40}=96\%$

The efficiency of 96% is attained under steady-state PV conditions. Over a simulated 24-h solar cycle with realistic irradiance patterns, the average efficiency remains around 93.5%. Table 3 compares Active ${X}^{2}$G-Boost converter with existing qZSN based topologies. The proposed design achieves the highest efficiency (96%) while operating at a low switching frequency (10 kHz), indicating reduced switching losses. It also offers a high voltage gain with a moderate component count, making it more compact and efficient than other converters that require higher frequencies or more components.

**Table 3:** Comparative evaluation of Active ${X}^{2}$G-Boost converter with qZSN topologies.

References	I	C	D	S	${f}_{s}$(kHz)	Voltage gain	Efficiency (%)
Rashid et al.^4^	4	5	4	2	25	$\frac{1}{1-2D}$	92.2
Nafari & Beiranvand^7^	3	4	6	1	50	$\frac{1}{{\left(1-D\right)}^{2}}$	85
Samadian et al.^14^	2	5	4	1	50	$\frac{2+n}{1-2D}$	95.5
Andrade et al.^20^	4	7	5	2	50	$\frac{3+2D}{1-2D}$	78
AbbasiAghdamMeinagh et al.^21^	2	6	6	3	40	$\frac{4}{1-2D}$	94.6
Caldeira et al.^22^	2	5	5	2	100	$\frac{3+3D}{1-3D}$	95
Proposed	2	4	5	1	10	$\frac{2D}{1-D}$	96

This demonstrates the advantage of the proposed converter in terms of performance, structural simplicity, and power quality for PV applications. Performance analysis of proposed Active ${X}^{2}$G Boost converter with recent advanced topologies is compared and summarized in Table 4. The proposed converter offers lower current stress, moderate switch voltage stress, and high voltage gain. It also achieves the lowest input current ripple, contributing to better EMI performance. With an efficiency of 96%, Active ${X}^{2}$G Boost converter with HSTM optimized PI controller outperforms other designs, demonstrating superior overall performance, reduced stress, and enhanced power quality.

**Table 4:** Comparison of Active ${X}^{2}$GBoost converter with recentconverter topologies.

References	Diode current stress	Diode voltage stress	Switch current stress	Switch voltage stress	Voltage gain	Input current ripple	Efficiency (%)
Zhang et al.^3^	$\frac{{I}_{o}}{1-D}$	$\frac{2{V}_{PV}}{1-D}$	$\frac{4{I}_{o}}{1-D}$	$\frac{{V}_{PV}}{1-D}$	$\frac{4}{1-D}$	$\frac{D{V}_{PV}}{L{f}_{s}}$	93.45
Rashid et al.^4^	$\frac{1}{1-2D}{I}_{r}$	${V}_{o}$	$\frac{2}{1-4{D}^{2}}{I}_{r}$	${V}_{o}$	$\frac{1}{1-2D}$	$\frac{D{V}_{PV}}{L{f}_{s}}$	92.2
Singh et al.^6^	${I}_{o}$	$\frac{(1-D)}{2(1+D)}{V}_{o}$	$\frac{(1+3D)}{2(1-D)}{I}_{o}$	$\frac{{V}_{o}}{2}$	$\frac{2(1+D)}{1-D}$	–	91.4
Nafari & Beiranvand^7^	–	${V}_{{C}_{3}}+\frac{{\Delta }_{{V}_{C3}}}{2}$	$\frac{2(2D+{D}^{2}{D}^{3}){I}_{o}}{{(1-D)}^{2}}$	$\left({V}_{{C}_{4}}+\frac{{\Delta }_{{V}_{C4}}}{2}\right)-\left({V}_{{C}_{3}}+\frac{{\Delta }_{{V}_{C3}}}{2}\right)$	$\frac{1}{{\left(1-D\right)}^{2}}$	–	85
Sun et al.^8^	$\frac{{P}_{o}}{\left(1-D\right){V}_{o}}$	$\frac{{V}_{o}}{2}$	$\frac{2nK{P}_{o}}{D(1-2D){V}_{o}}$	$\frac{1}{2nK}{V}_{o}$	$\frac{2nk}{1-2D}$	$\frac{KD(1-D){V}_{PV}}{2(1-2D)L{f}_{s}}$	94
Sun et al.^9^	$\frac{{I}_{o}\sqrt{2\pi }wtT}{4}$	$\frac{{V}_{o}(2+2n)}{3+D+2n}$	–	$\frac{{V}_{o}}{3+D+2n}$	$\frac{3+D+2n}{1-D}$	$\frac{(1-2D)\Delta {i}_{{L}_{m}}}{1-D}$	95.4
Proposed converter	$\frac{2D}{1-D}\cdot \frac{{V}_{PV}}{{R}_{load}}$	${V}_{PV}\cdot \frac{2D}{1-D}$	${I}_{o}\left(\frac{1-D}{2D}\right)$	${V}_{PV}\cdot \frac{2D}{1-D}$	$\frac{2D}{1-D}$	$\frac{2{V}_{PV}\cdot D\cdot {T}_{S}}{L}$	96

**Fig. 30:**
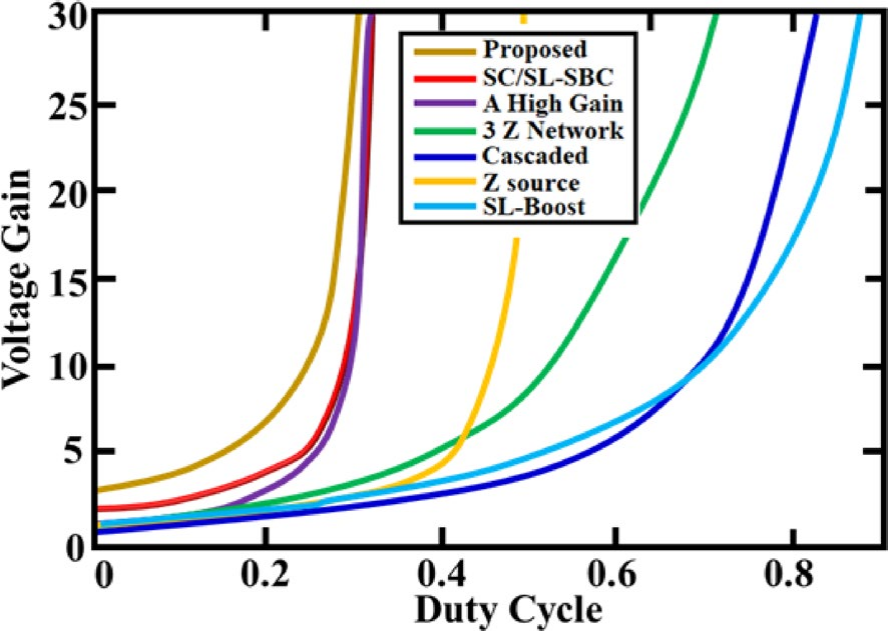
Voltage gain evaluation of Active ${X}^{2}$G Boost converter against conventional designs.

Figure 30 presents voltage gain versus duty cycle comparison for various converter topologies. Active ${X}^{2}$G Boost converter achieves the highest voltage gain at lower duty cycles. surpassing conventional topologies such as Z-Source, SL-Boost and Cascaded converters. Steep gain curve enables high output with reduced switching stress, making it ideal for efficient PV based EV applications. These results demonstrate the improved performance of the converter under low-duty and high-gain operating conditions.

**Fig. 31:**
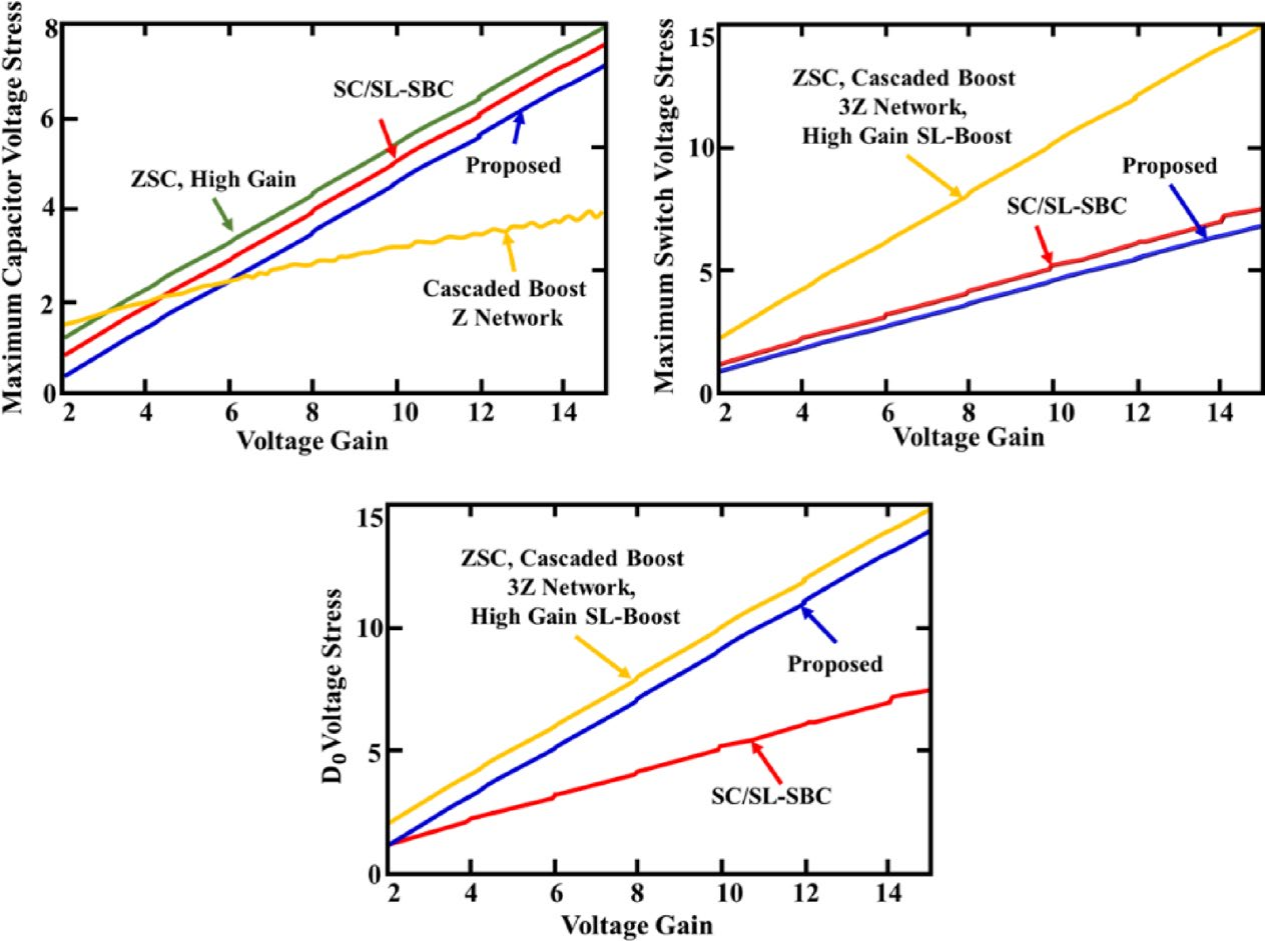
Comparative stress analysis of capacitor, switch and output diode with voltage gain.

Comparative stress analysis of capacitor, switch and output diode with voltage gain relative to voltage gain is shown in Fig. 31. The proposed converter consistently exhibits lower voltage stress than Z-source converter and CBC especially at higher voltage gains. While Switched Capacitor (SC) and Switched Inductor (SL) networks within a boost converter topology also shows good performance, the proposed design strikes a better balance between gain and reduced stress, ensuring enhanced reliability and efficiency in high-gain applications.

Table 5 presents a comparative analysis of Genetic Algorithm-tuned PI (GA-PI), Particle Swarm Optimization-tuned PI (PSO-PI) and the proposed HSTM-PI based on key dynamic performance metrics. The GA-PI controller exhibits a rise time of 0.231 s and settling time of 0.2988 s, along with a relatively high overshoot of 8.432%. The PSO-PI controller improves significantly, reducing rise time to 0.0125 s and settling time to 0.1285 s, while lowering overshoot to 7.7829%. The proposed HSTM-PI controller outperforms both, achieving the fastest rise time of 0.0113 s, the shortest settling time of 0.1096 s and the lowest overshoot of 5.74%.

**Table 5:** Comparative performance analysis of conventional and the proposed HSTM-PI based tuning methods.

Techniques used	Rise time (s)	Settling time (s)	Overshoot (%)
Conventional GA-PI^33^	0.0146	0.2988	8.432
Conventional PSO-PI^34^	0.0125	0.1285	7.7829
Proposed HSTM-PI controller	0.0113	0.1096	5.74

**Fig. 32:**
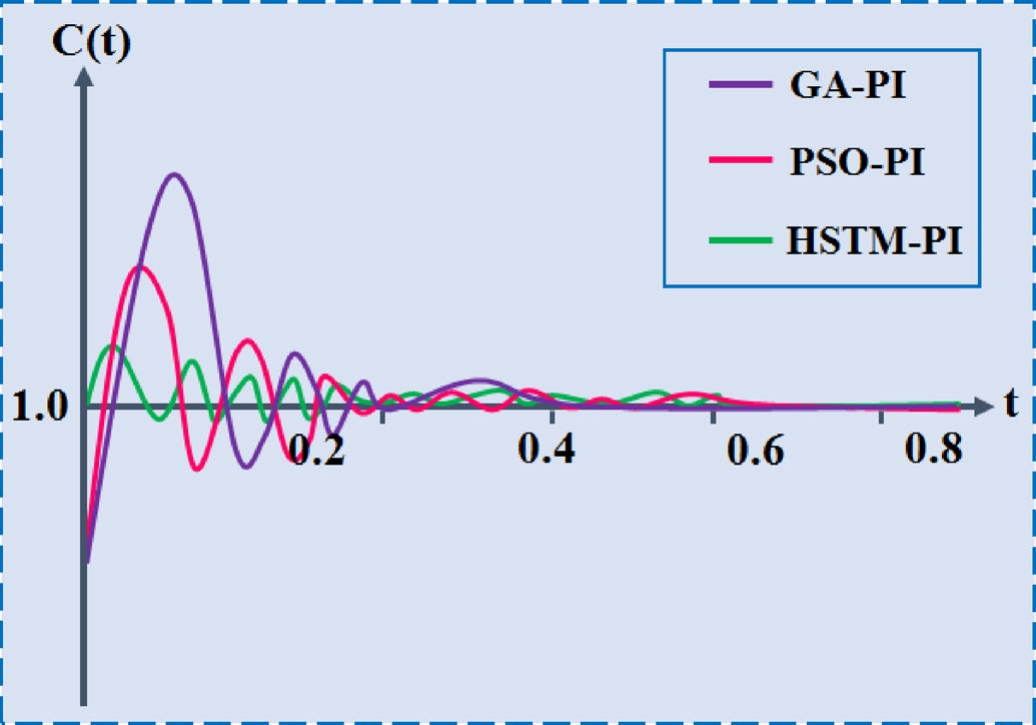
Step response performance analysis of conventional and proposed tuning methods.

The plot in Fig. 32 illustrates the dynamic response of three different PI control strategies to a unit step input. The GA-PI controller exhibits the largest overshoot and the slowest settling time, indicating less stability and slower dynamic response. The PSO-PI controller shows improved performance with a faster rise and lower overshoot compared to GA-PI, but it still displays noticeable oscillations. In contrast, the HSTM-PI controller achieves the best performance, with minimal overshoot, fastest settling time and superior damping of oscillations.

In high-power or fast EV charging (DCFC) scenarios, the proposed system faces certain limitations primarily related to thermal management, EMI and component stress. Although the single-switch Active X^2^G Boost converter is optimized for efficiency, the elevated current levels in fast charging operations can cause increased conduction losses and heating in inductors, capacitors and switching devices, potentially affecting long-term reliability. These limitations can be addressed in future; moreover, this work can be extended to address unbalanced load conditions and incorporate vehicle-to-grid (V2G) operations. Furthermore, future investigations will consider optimizing the controller design for higher efficiency under dynamic operating conditions, integrating renewable energy sources for sustainable EV charging, and evaluating system performance in real-time hardware-in-the-loop (HIL) platforms. These directions will further enhance the applicability of the proposed converter in practical smart grid and e-mobility scenarios.

## Conclusions

The research work carried out in this paper successfully presents the design, modelling, and validation of a novel Active X^2^G Boost converter for PV powered EV charging systems. Through a combination of a qZSN and a voltage multiplier stage, the proposed converter achieves high voltage gain. The integration of the HSTM-PI controller ensures robust voltage regulation under both steady-state and dynamically varying environmental conditions. Extensive MATLAB/Simulink simulations and real-time hardware implementation validate the converter’s performance. Under constant solar conditions, the system delivers a stable DC-link voltage with minimal ripple, while the battery charging profile remains well-regulated. In dynamic scenarios, the converter maintains output stability despite sudden changes in irradiance. During PV disconnection events, the system efficiently transitions to battery support mode via the bidirectional DC–DC converter, ensuring uninterrupted power flow and grid synchronization. The system achieves a peak efficiency of 96% compared with several existing topologies in both gain and performance metrics.

## Data Availability

The datasets used and/or analysed during the current study available from the corresponding author on reasonable request.
